# The Consequences of Failing to Act: Examining the Importance of Perceived Employee-Directed Managerial Inaction

**DOI:** 10.1177/15480518251352592

**Published:** 2025-07-03

**Authors:** Christine C. Hwang, Laurie J. Barclay, Daniel L. Brady

**Affiliations:** 1 Lang School of Business & Economics, 3653 University of Guelph, Guelph, Ontario, Canada; 2 Lazaridis School of Business & Economics, 8431 Wilfrid Laurier University, Waterloo, Ontario, Canada

**Keywords:** perceived employee-directed managerial inaction, fundamental social dilemma, negative gossip, resistance behavior, citizenship behavior

## Abstract

With increasing momentum to hold managers accountable for their failure to act in response to aversive employee experiences, it is critical to develop a conceptual and theoretical understanding of this phenomenon. We introduce *perceived employee-directed managerial inaction* to capture employees’ perceptions that their manager failed to act in response to an aversive event that they experienced despite a perceived duty or obligation for the manager to do so. Drawing on the fundamental social dilemma, we propose that perceived employee-directed managerial inaction is negatively associated with employees’ perceptions that their manager is trustworthy, which can prompt detrimental outcomes for managers (withdrawal of manager-directed citizenship behavior, resistance behavior, and negative gossip about the manager) as well as for employees (lower psychological well-being). To investigate *what* perceived employee-directed managerial inaction is as well as *why* and *how* it can impact managers and employees, we develop a conceptualization of this construct, validate a measure, and test our theoretical model using an experiment and two multi-wave surveys. Contributions include answering calls to consider the importance of inactive and undesirable event-based responses, conceptually defining perceived employee-directed managerial inaction, and providing a validated measure to stimulate empirical research for this theoretically and practically important phenomenon. We also showcase *why* and *how* perceived employee-directed managerial inaction can have negative implications, including how this can inform employees’ generalized perceptions of managers. Overall, we highlight the importance of recognizing that perceived employee-directed managerial inaction is not benign but rather an undesirable response that can negatively impact managers and employees.

‘*It is not only what we do, but also what we do not do, for which we are accountable*.’

– Molière

Recently, the media has been awash with stories reflecting the growing momentum to hold organizations legally and ethically accountable for managers who “did nothing” in response to knowing about harmful behaviors toward their employees (MSN, 2021). For example, a lawsuit was launched to hold a district liable for the inaction of its head coach, who allegedly failed to take action after team members discussed hazing a particular team member in front of him (Good, 2022). Indeed, these lawsuits have become a “watershed moment for the business world” (Iyengar, 2021). Despite the clear practical relevance of managerial inaction within organizations, this has been underemphasized in the scholarly literature. Instead, scholarly attention has been focused on examining managers’ positive or negative actions (i.e., behaviors). However, scholars have strongly criticized the literature for “confusing action with inaction” and have pointed to the theoretical importance of shifting focus to develop a deeper understanding of inactive responses (Cropanzano et al., 2017, p. 16). This includes calling for research that develops new constructs that capture inactive responses as well as considering that a *lack of action* can be “undesirable” and impactful.

Answering these calls, we introduce *perceived employee-directed managerial inaction* to reflect an employee's perception that their manager failed to uphold managerial duties and/or responsibilities to act in response to the employee experiencing an aversive event. Drawing on the fundamental social dilemma (e.g., Lind, 2001), we argue that perceived employee-directed managerial inaction can detract from employees’ perceptions that their manager is trustworthy, which can prompt employees to protect themselves from the manager. This can have significant costs for managers by eliciting employee behaviors that can be dysfunctional for managers (i.e., withdrawing citizenship behavior, engaging in resistance behavior, and engaging in negative gossip about the manager) and costs for employees by detracting from their psychological well-being. Figure 1 displays our theoretical model.

**Figure 1.**
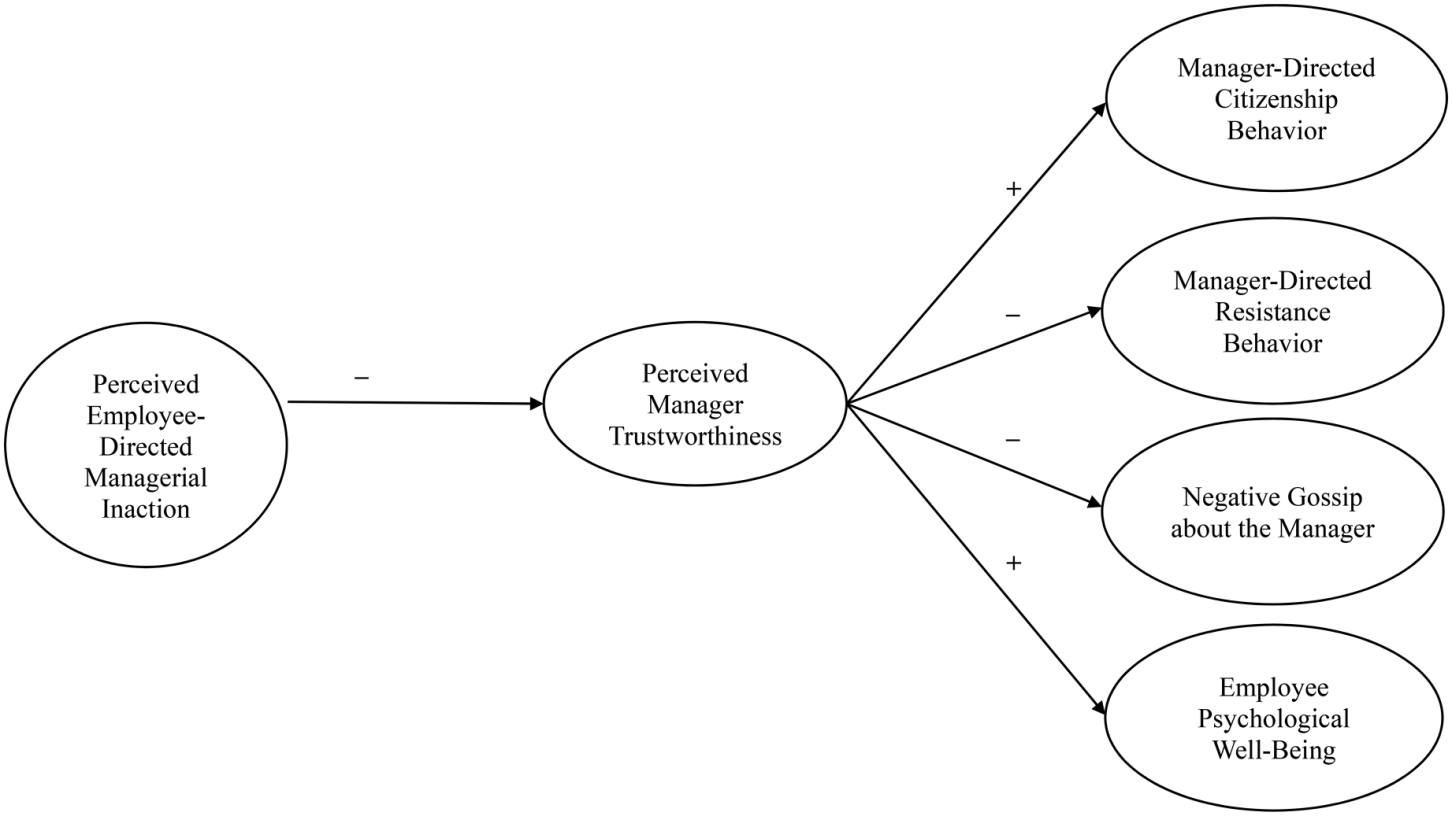
Theoretical model.

We contribute to the literature in three ways. First, by introducing perceived employee-directed managerial inaction to the literature, we answer scholarly calls to tackle practically significant issues (e.g., Tsui, 2021) and explore omissions of behavior that can be inactive/undesirable (e.g., Cropanzano et al., 2017). We also expand recent arguments in the leadership literature related to the importance of focusing on what managers “do” to impact others (e.g., Fischer et al., 2023) to also consider how managers’ failure to engage in behavior may also be impactful. More precisely, we showcase the theoretical importance of recognizing that managers’ responses to specific events do not have to be active to be consequential. Taken together, we introduce and provide a conceptual definition of perceived employee-directed managerial inaction as well as develop and validate a measure to enable further empirical research related to this theoretically and practically important construct.

Second, while an extensive literature has examined how generalized tendencies (e.g., leadership styles) can impact employees, there have been emerging calls to examine the impact of single discrete events (e.g., Liu et al., 2023). Indeed, recent calls have been issued for adopting an event-based approach to better understand the impact of discrete workplace events (e.g., Morgeson et al., 2015), including the effects of discrete workplace events on generalized perceptions (e.g., Kiefer et al., 2022). Adopting an event-based approach can be especially important in light of arguments that effective management and leadership happen on an everyday basis as specific events occur (e.g., Morgeson & DeRue, 2006). We argue that perceived employee-directed managerial inaction (i.e., an inactive and undesirable event-based response) can impact employees’ perceptions related to their manager's trustworthiness (i.e., a generalized perception), even when considering their manager's generalized leadership styles. In doing so, we explore how a single event of perceived employee-directed managerial inaction can have detrimental implications by impacting employees’ broader and longer-term evaluations of managers’ trustworthiness (i.e., generalized perceptions of managers).

Third, we identify perceived trustworthiness as a mechanism that can illuminate *why* perceived employee-directed managerial inaction can have detrimental effects and investigate *how* perceived employee-directed managerial inaction can negatively impact managers and employees. By detracting from perceived trustworthiness, we argue that perceived employee-directed managerial inaction can prompt employees to engage in behaviors to protect themselves from the manager. This includes withdrawing positive behavior (i.e., manager-directed citizenship behavior) from their manager and engaging in negative behaviors toward their manager (i.e., engaging in resistance behavior and negative gossip about their manager)—behaviors that can undermine managerial effectiveness. By detracting from perceived trustworthiness, perceived employee-directed managerial inaction may also undermine employee well-being. By outlining and empirically examining this process, we provide evidence that perceived employee-directed managerial inaction is indeed an undesirable response while also identifying why and how it may have detrimental effects for managers and employees.

## Examining Event-Based Inactive Managerial Responses

Within the literature, there has been a strong emphasis on how managers generally behave over time and situations. For example, an extensive literature on leadership styles has indicated that some managers may generally engage in individualized consideration (e.g., transformational leadership) whereas others may generally avoid intervening in situations (e.g., laissez-faire leadership; see Bass & Avolio, 1990). These more global evaluative assessments across time and situations are referred to as “entity” perceptions and can be informative for capturing how employees generally perceive their managers (see Cropanzano et al., 2001). However, employees may also hold “event” perceptions that are related to how employees perceive their manager in relation to a specific occurrence or event. As Cropanzano et al. (2001, p. 189) outline: “it is one thing to say ‘my supervisor treated me fairly during my last feedback session’ [event perception], and quite another to say ‘my supervisor is a fair person’ [entity perception]”. Indeed, research has demonstrated that this distinction is critical because entity and event perceptions initiate distinct processes. For example, employees’ entity perceptions can be influential for how employees may generally behave across time and situations towards their managers whereas employees’ event perceptions can be influential to how employees respond to a specific situation, including the behaviors that are elicited to manage the particulars of the situation (e.g., Kiefer et al., 2022). As such, an event-based approach creates the opportunity to examine how specific managerial responses can impact employees as well as how specific events may impact employees’ generalized perceptions of their managers.

While scholars have long recognized that managers’ *behavior* can impact employees, an event-based approach also illuminates the importance of considering managers’ *inactive* responses to situations—a point that is often overlooked when generalized tendencies (entity-based approaches) are the focus (Cropanzano et al., 2017). Applying an event-based approach, Folger and Cropanzano (2001) argued that people can be held responsible for withholding helpful acts and noted that “nonintervention is greatly in need of future research” (p. 14). Relatedly, Cropanzano et al. (2017, p. 17) argued that responses can be understood in terms of whether they are *active* or *inactive* and criticized the current practice of conflating action with inaction by treating “the absence of the variable – roughly a low score on the instrument—as the opposite manifestation of the variable in question.” Instead, these authors argued it is important to distinguish between active versus inactive constructs as well as identify whether these constructs are desirable or undesirable.

For the active/inactive dimension, active responses reflect engaging in a particular behavior whereas inactive responses reflect withholding or not engaging in a particular behavior (Cropanzano et al., 2017). For the desirable/undesirable dimension, desirable constructs are hedonically positive and may occur when something positive is provided or something negative is withheld. By contrast, undesirable constructs are hedonically negative and may occur when something positive is withheld or something negative is provided. When these two dimensions are crossed, four quadrants emerge. Whereas the literature has focused on active/desirable managerial behaviors (e.g., support, justice) and active/undesirable managerial behaviors (e.g., social undermining, injustice), inactive/desirable and inactive/undesirable behaviors have received less attention.^1^ This is a critical gap since inactive constructs are theoretically distinct and may also impact employees. As an example, consider how a manager may actively support an employee (e.g., provide reassurance) or actively undermine an employee (e.g., criticize an employee). However, as Cropanzano et al. (2017, p. 19) outline, “an individual can withhold support without being abusive or can refrain from antisocial conduct without becoming an altruist” suggesting that it is critical to challenge the assumption that “the absence of a positive is equal to the presence of a negative or vice versa”. Similarly, a manager can engage in behavior to uphold (active, desirable) or violate (inactive, undesirable) their managerial duties, such as taking action to uphold safety protocols or taking action that violates safety protocols in response to an employee experiencing an unsafe experience, respectively. However, managers can also fail to engage in behaviors related to their managerial duties (inactive, undesirable), such as withholding or not engaging in behaviors in response to the employee's unsafe experience. Recognizing this distinction suggests the importance of considering *inactive* responses (i.e., withholding or failing to engage in a particular behavior).

## Perceived Employee-Directed Managerial Inaction

Cropanzano et al. (2017, p. 20) argue that identifying “new constructs” that reflect inactive and undesirable responses to fill this “missing cell” is not only critical because these have been underemphasized in the literature but also because inactive/undesirable responses may have impactful consequences. Building on this foundation, we introduce *perceived employee-directed managerial inaction* to reflect employees’ event-based perceptions of an inactive/undesirable managerial response. More precisely, we define perceived employee-directed managerial inaction as *an employee's perception that a manager failed to engage in appropriate behaviors to prevent and/or address an employee's aversive experience when there was a perceived obligation and/or expectation to do so.* There are several elements of this conceptual definition that are important to highlight. Perceived employee-directed managerial inaction is *event-based* rather than a general tendency to be negligent across situations. This distinguishes perceived employee-directed managerial inaction from generalized entity perceptions that occur across time and situations (see Cropanzano et al., 2001), including leadership styles (i.e., generalized patterns of attitudes that leaders hold and generalized patterns of behaviors that they exhibit; Anderson & Sun, 2017). For example, perceived employee-directed managerial inaction is different from passive leadership styles such as amoral management (i.e., managers’ consistent failure to respond to issues with ethical implications). Indeed, amoral management is “not a one-time occurrence of avoiding ethics; rather, ‘avoidance’ is a regular management practice with respect to ethics and is likely to continue until leaders change their management styles or are replaced” (Quade et al., 2022, p. 276). As an event-based phenomenon, managers with different leadership styles can engage in perceived employee-directed managerial inaction (i.e., inaction may occur regardless of generalized leadership style).

Consistent with previous research indicating the importance of specifying the target of a response (e.g., Bennett & Robinson, 2000), perceived employee-directed managerial inaction is conceptualized as being *targeted towards employees*. While other forms of managerial inaction may occur (e.g., a failure to respond to financial losses, threats to the organization, or customer complaints), these have different targets and are conceptually distinct from employee-directed managerial inaction. Further, previous research indicates that constructs may be conceptualized differently depending on perspective (e.g., manager, employee, third parties; Long, 2016). As such, we note that perceived employee-directed managerial inaction emphasizes the *employee's* perspective. This construct is also *perceptual* in nature (i.e., in the ‘eye of the beholder’), such that it captures employees’ *subjective* evaluations of their managers’ responses. Recognizing that perceived employee-directed manager inaction is subjective is important because employees may hold differing perceptions related to whether there is an obligation or expectation for managers to act and these subjective perceptions are critical drivers of behavior (Greenberg et al., 1991).

Three conditions must be met to qualify as perceived employee-directed managerial inaction. First, perceived employee-directed managerial inaction is conceptualized as an inactive and undesirable event-based response that occurs in response to an employee experiencing or having the potential to experience a harmful or unpleasant situation at work (i.e., an event). Consistent with theory (e.g., Cropanzano et al., 2017) and empirical evidence (e.g., Lindquist et al., 2016) indicating that events can either be desirable or undesirable, perceived employee-directed managerial inaction is conceptualized as an undesirable response because it reflects employees’ perceptions that the manager failed to fulfill managerial responsibilities, such as duties to care for employee well-being (Petrin, 2012) or maintain a safe and ethical environment (Brown et al., 2005). That is, perceived employee-directed managerial inaction withholds a positive behavior from employees related to the fulfillment of managerial duties. Said differently, this is an *inactive* response because managers *failed to act* (i.e., withheld or did not engage in positive behaviors) when an employee was exposed to potential harm (e.g., when a manager fails to step in after witnessing an employee being subjected to incivility).

Second, the manager is *reasonably* expected to be aware of the possibility that employees may have an aversive experience. If a manager is not reasonably expected to be aware of the potential for harm to the employee, then they are not abdicating their managerial duties. For example, employees are unlikely to blame a manager for their lack of action if the manager was not present when they were being harmed. However, if the manager has witnessed or been informed of the situation, their lack of action satisfies the second condition for perceived employee-directed managerial inaction.

Third, the employee's perception that the manager has not acted to prevent and/or address the issue despite being aware violates the employee's expectations related to the moral or social norms, duties, or responsibilities attached to the managerial role. More precisely, managers have obligations to discipline inappropriate behaviors as well as the *duty of care* to protect employees from workplace harm (Kahan, 1989). Managers are also required to react to and proactively take reasonable steps to prevent or manage potential harm to employees (Stickley, 2017). Thus, the perceived failure of a manager to prevent or respond to employees’ aversive experiences violates these managerial duties and obligations. This is consistent with the notion that “people are most likely to be held responsible for sins of omission when there is a clear normative expectation that a certain beneficial action be taken” (Folger & Cropanzano, 2001, p. 14).

## The Impact of Perceived Employee-Directed Managerial Inaction on Outcomes

To investigate the effect of perceived employee-directed managerial inaction on employees, we draw on the *fundamental social dilemma* (Lind, 2001), which reflects a tension that can emerge from specific events that involve employees’ interactions with their managers. More precisely, the fundamental social dilemma is a psychological process that can unfold when people recognize that they can benefit from behaving cooperatively with others, but also recognize that doing so can also “run the risk that those others will take more than they give” (Lind, 2001, p. 62). As such, people are motivated to resolve this dilemma by ensuring that their investments are not exploited by others. Applied to perceived employee-directed managerial inaction, cooperating with managers provides opportunities for gaining positive outcomes. Yet, it can also create the potential for exploitation (e.g., managers taking employees’ input without reciprocating). As such, employees are motivated to assess their manager's trustworthiness because this provides information about how employees should navigate their relationship with the manager (e.g., to prevent exploitation). Building on this, we argue that perceived employee-directed managerial inaction can give rise to the fundamental social dilemma by raising concerns about managerial trustworthiness and prompting employees to engage in behaviors to protect themselves. We outline our theoretical rationale for these relationships below. In doing so, we also demonstrate the importance of an event-based approach by examining how perceived employee-directed managerial inaction, as a discrete event, can influence employees’ generalized perceptions of a manager as an entity (i.e., perceived manager trustworthiness).

### Perceived Employee-Directed Managerial Inaction and Manager Trustworthiness

Managers are responsible for maintaining a safe and ethical work environment, protecting their employees from harm, and setting appropriate norms (e.g., Brown et al., 2005). However, by definition, perceived employee-directed managerial inaction reflects a specific instance where the manager is perceived to have failed to uphold their managerial responsibilities. Building on the notion that negative discrete events can prompt employees to assess their manager's trustworthiness (e.g., Lind, 2001), we argue that perceiving that the manager has failed to act can prompt employees to question the manager's capability to carry out their managerial responsibilities and whether the manager intends to uphold moral or social norms. Said differently, perceived employee-directed managerial inaction can give rise to the fundamental social dilemma by prompting employees to perceive the manager as less trustworthy (i.e., the manager is lacking competencies, the motivation to “do good”, and/or integrity; Mayer et al., 1995). Thus, we predict that perceived employee-directed managerial inaction is negatively associated with perceived manager trustworthiness because the perceived failure of the manager to act when there was an expectation to do so can create concerns about whether the manager *can* and *will* act in a trustworthy manner moving forward (Colquitt et al., 2007).

*Hypothesis 1*: Perceived employee-directed managerial inaction is negatively related to employees’ perceptions of manager trustworthiness.

### Perceived Employee-Directed Managerial Inaction and Self-Protective Behaviors

Building on our argument that perceived employee-directed managerial inaction can detract from perceived manager trustworthiness, we argue that this can raise concerns about whether the manager is likely to exploit the employee's willingness to cooperate and thus motivate the employee to protect themselves from potential exploitation. More precisely, the fundamental social dilemma suggests that perceiving low manager trustworthiness may prompt employees to focus on self-protection to reduce the potential or risk for exploitation by their manager. This desire for self-protection may manifest as enacting less positive and more negative discretionary behaviors toward the manager to reduce the potential for exploitation as well as being less likely to behave cooperatively towards the manager (e.g., looking for opportunities to contribute to managers’ needs and goals) to avoid investing in a relationship that runs the risk that these investments may be exploited (e.g., Lind, 2001; McAllister, 1995).

Consistent with the fundamental social dilemma, we examine manager-directed citizenship behavior because employees can withdraw this positive discretionary behavior to protect themselves without risk of penalty (e.g., Podsakoff et al., 2000). Given that perceived employee-directed managerial inaction can motivate employees to protect their interests from a manager that is not perceived as trustworthy, we argue that perceived employee-directed managerial inaction can prompt employees to withdraw manager-directed citizenship behavior to limit how much their manager can “take” from them. Said differently, perceived employee-directed managerial inaction may prompt employees to withdraw manager-directed citizenship behavior because they are less likely to perceive that their manager is trustworthy and therefore need to protect themselves from potential exploitation.

*Hypothesis 2*: Perceived employee-directed managerial inaction is negatively related to manager-directed citizenship behavior (H2a) via perceived manager trustworthiness (H2b).

The fundamental social dilemma may also prompt employees to engage in behaviors that actively protect themselves and reduce the manager's influence over them (Lind, 2001). Building on this premise, we propose that perceived employee-directed managerial inaction can prompt employee resistance behavior (i.e., behaviors that reflect non-compliance rather than direct challenge or deviance, such as resisting managers’ requests; Tepper et al., 1998). Resistance behavior can enable employees to protect themselves from potential exploitation (e.g., by reducing their contributions) without placing them at further risk, especially since these behaviors can be difficult for managers to detect and punish (Tepper et al., 1998).

*Hypothesis 3*: Perceived employee-directed managerial inaction is positively related to employee resistance behavior (H3a) via perceived manager trustworthiness (H3b).

Perceived employee-directed managerial inaction may also prompt employees to protect themselves by engaging in negative gossip about the manager (i.e., informal and evaluative talk that occurs when the manager is not present; Brady et al., 2017). Negative gossip about the manager can serve as a form of social control, such that sharing negative information can help protect the employee's own reputation while also indicating that others should also protect themselves from the manager (e.g., Feinberg et al., 2012). As such, we propose that perceived employee-directed managerial inaction can prompt negative gossip towards the manager to protect the employee and others from the manager and this relationship can be explained by concerns about the manager's perceived trustworthiness.

*Hypothesis 4*: Perceived employee-directed managerial inaction is positively related to employees’ negative gossip about the manager (H4a) via perceived manager trustworthiness (H4b).

### Perceived Employee-Directed Managerial Inaction and Psychological Well-Being

Beyond initiating behaviors in response to the manager, we propose that perceived employee-directed managerial inaction may also directly impact employees. By perceiving that the manager has failed to fulfill their duties, perceived employee-directed managerial inaction may detract from employees’ confidence that their manager will help them with job-related difficulties, demonstrate care, or behave with integrity in the future (e.g., Chughtai et al., 2015). This may undermine employee well-being by making employees feel the need to avoid being vulnerable with their manager and that they must instead divert energy toward protecting themselves (e.g., Dirks & Ferrin, 2002). Given that having a trusting relationship with one's manager is critical for workplace well-being (e.g., Helliwell & Huang, 2011), we propose that perceived employee-directed managerial inaction is negatively related to employees’ well-being via perceived manager trustworthiness.

*Hypothesis 5*: Perceived employee-directed managerial inaction is negatively related to employee well-being (H5a) via perceived manager trustworthiness (H5b).

## Overview of Studies

We use a multi-method approach to test our hypothesized model with samples of employed adults. To enhance internal validity and reduce potential endogeneity concerns (see Antonakis et al., 2010), Study 1 uses an experiment to manipulate perceived employee-directed managerial inaction. Next, we develop and validate a measure of perceived employee-directed managerial inaction. To enhance external validity, Studies 2 and 3 test our full theoretical model in the context of ongoing working relationships with three-wave surveys. We also provide incremental validity evidence for perceived employee-directed managerial inaction beyond theoretically related constructs (Study 2) and generalized leadership styles (Study 3). All studies were approved by institutional research ethics boards. We preregistered Studies 1 and 3 hypotheses, manipulations (Study 1), and analytic strategy. We followed recommendations to screen for careless responders and set inclusion criteria a priori (e.g., Goldammer et al., 2020). Study 1 is available at: https://osf.io/84srf/?view_only=0aa5f4895eaa45af8f163731150cd3e6; Study 3 is available at: https://osf.io/fv3j4/?view_only=7c0414c562e840abac7f6c42dd6792a0).

## Study 1

Study 1 tests the hypothesized effects of perceived employee-directed managerial inaction on perceived manager trustworthiness and manager-directed behaviors. We used an experimental vignette to manipulate the level of perceived employee-directed managerial inaction to establish temporal ordering with perceived manager trustworthiness and rule out alternative explanations, thereby enhancing internal validity (see Aguinis & Bradley, 2014).

### Sample and Procedure

Employed adults from the United States, Canada, and United Kingdom were recruited using Prolific and compensated for their online participation (£.87). A power analysis for a mediation with medium strength correlations (*r* = .30), a 95% confidence interval, and a desired power of .80 (see Schoemann et al., 2017) indicated that a sample of 234 was needed. We recruited a slightly larger sample to account for missing data and careless responders.

In total, 259 individuals qualified for participation and finished the study. Following our preregistered exclusion criteria, data was excluded from 5 participants who requested their data not be used, 17 for incorrect responses to the manipulation check, and 1 for having a univariate outlier (> 3 *SD* from the mean). All remaining participants accurately responded to at least two of the three attention checks and therefore met this a priori inclusion criterion. The final sample was comprised of 236 participants (50.4% female; mean age = 36.17 years, *SD* = 9.96; mean organizational tenure = 5.50 years, *SD* = 6.27; 81.4% identified as Caucasian, 7.2% as Asian, and 4.2% as Black or African American). Participants were employed in 22 different industries, including business and financial operations (16.1%), education (14.8%), healthcare (13.6%), and government (7.6%).

Participants read the following scenario:

You are an employee at a local insurance company. You have been at this company for three years and you work full-time. Your manager assigned you to work on a project team with several other employees. This morning, you were presenting a recommendation and implementation plan for a client's project at an internal meeting. After your presentation, a project team member mocked and attacked your idea, saying that “your recommendations are absolutely useless” and “I can’t believe you thought this would work – you must be really dumb.”

Participants were randomly assigned to one of three conditions: perceived employee-directed managerial inaction, perceived employee-directed managerial action, or control. In the perceived employee-directed managerial inaction condition, participants read that the manager did not do anything in response to the project team member's behavior (“Your manager saw the situation and did not do anything to address the project team member's bad behavior.”; *n* = 86). In the perceived employee-directed managerial action condition, the manager addressed the project team member's behavior (“Your manager saw the situation and acted to address the project team member's bad behavior.”; *n* = 75). In the control condition, participants read that the manager was unaware of the project team member's behavior (“Your manager did not see the situation and is therefore not aware of the project team member's bad behavior”; *n* = 75).^2^

### Measures

To maintain the temporal ordering of our variables, we presented the measure for the mediator first, followed by the measures for the dependent variables. We asked participants to answer the manipulation check after the dependent variables to avoid any unintentional impact on the variables of interest in the model (see Hauser et al., 2018).

*Perceived manager trustworthiness* was assessed using Mayer and Davis (1999) scale that measures the three sub-dimensions of perceived trustworthiness: ability (6-items; e.g., “My manager is very capable of performing their job”), benevolence (5-items; “My manager is very concerned about my welfare”), and integrity (6-items; e.g., “Sound principles seem to guide my manager's behavior”). The response anchors ranged from 1 = *strongly disagree* to 5 = *strongly agree*. Perceived manager trustworthiness was created by averaging the items within each sub-dimension, then using the three sub-dimensions as indicators for a latent perceived manager trustworthiness factor.^3^

*Manager-directed citizenship behavior* was assessed using Dalal et al.'s (2009) 6-item scale. Participants were asked to “Please indicate the extent to which you are likely to engage in the following behaviors towards your manager in scenario” (e.g., “(I will) try to help my manager”; 1 = *not at all* to 7 = *to a great extent*).

*Resistance behavior* was assessed using Tepper et al.'s (1998) 6-item scale. Participants were asked to indicate how likely they would be to engage in behaviors if their manager in the scenario made a request of them (e.g., “I will refuse to carry out the request from my manager”; 1 = *strongly disagree* to 5 = *strongly agree*).

*Negative gossip about the manager* was assessed using Brady et al.'s (2017) 5-item scale (e.g., “I will question the manager's abilities while talking to a coworker”; 1 = *not at all* to 7 = *to a great extent*).

Our manipulation check asked participants to “Choose the option that best describes the manager's response to the project team member's behavior in the scenario. The manager…”. Response options were “Failed to take action (i.e., did not address the project team member's behavior)”; “Addressed the project team member's behavior”; “Did not see the situation and is therefore not aware of the project team member's behavior”; and “Unsure”. Consistent with our pre-registration criteria noted above, 17 participants failed to correctly identify their condition and were excluded from the sample prior to our analyses.

### Results

We first conducted a confirmatory factor analysis (CFA) using Mplus version 8.1 to assess the factor structure of the measurement model (Muthén & Muthén, 2018). We began by testing a 4-factor model that included perceived manager trustworthiness, manager-directed citizenship behavior, resistance behavior, and negative gossip about the manager. Perceived manager trustworthiness was modeled with three indicators, which corresponded to the three dimensions of trustworthiness (i.e., ability, benevolence, and integrity; Mayer & Davis, 1999). We assessed model fit using Hu and Bentler's indices, which indicate that good model fit has been reached if the CFI is above .95, the RMSEA is less than .06, and the SRMR is below .08. While the CFI and SRMR met the criteria for good fit, the RMSEA was acceptable; χ^2^(164) = 341.463, *p* < .001, CFI = .950, RMSEA = .071, SRMR = .042. Before testing our hypotheses, we also compared the model fit of the 4-factor model to alternative factor structures. The 4-factor model had significantly better fit than all possible 3 factor models (smallest Δ*χ*^2^(3) = 215.78, *p* < .001), supporting the distinctiveness of the variables.

Table 1 shows the descriptive statistics, reliabilities, and correlations. To focus on the theoretical distinction between inaction versus action, our main analyses examine the perceived employee-directed managerial inaction versus action conditions (see supplemental analyses for perceived employee-directed managerial inaction versus the control condition).

**Table 1.** Study 1: Means, Standard Deviations, Zero-Order Correlations, and Reliabilities.

Variables	*M*	*SD*	1	2	3	4	5	6
1. Perceived employee-directed managerial inaction (vs. action)^a^	.53	.50	–					
2. Perceived employee-directed managerial inaction (vs. control)^b^	.53	.50	–	–				
3. Perceived manager trustworthiness	3.15	1.05	−.77***	−.64***	(.98)			
4. Manager-directed citizenship behavior	3.11	1.01	−.72***	−.59***	.79***	(.94)		
5. Resistance behavior	2.13	.82	.30***	.18*	−.37***	−.46***	(.87)	
6. Negative gossip about the manager	3.08	1.53	.46***	.33***	−.48***	−.46***	.55***	(.92)

*Note. N* = 236. Reliabilities are displayed in parentheses.

a Perceived employee-directed managerial inaction (vs. action) is the IV that contrasts action (coded as 0) with inaction (coded as 1); *n* *=* *161.*

b Perceived employee-directed managerial inaction (vs. control) is the IV that contrasts control (coded as 0) with inaction (coded as 1); *n* *=* *161.*

*p < .05. **p < .01. ***p < .001.

Hypothesis 1 predicted that perceived employee-directed managerial inaction is negatively related to perceived manager trustworthiness. To test this hypothesis, we conducted an independent samples t-test between the perceived employee-directed managerial inaction and action conditions. Given that Levene's test for the equality of variances was significant, *F*(1, 159) = 4.19, *p* = .042, equal variances were not assumed. As predicted, perceived manager trustworthiness was significantly lower in the perceived employee-directed managerial inaction condition (*M* = 2.18, *SD* = .80) than in the perceived employee-directed managerial action condition (*M* = 3.94, *SD* = .64), *t*(158.10) = 15.47, *p* < .001, *d* = 2.41. H1 was supported.

Results indicated that manager-directed citizenship behavior was significantly lower for perceived employee-directed managerial inaction (*M* = 2.23, *SD* = .87) than action (*M* = 3.83, *SD* = .61), *F*(1, 159) = 8.62, *p* = .004, *t*(152.44) = -13.55, *p* < .001, *d* = 2.09. Resistance behavior was significantly higher for perceived employee-directed managerial inaction (*M* = 2.38, *SD* = .84) than action (*M* = 1.88, *SD* = .78), *t*(159) = 3.95, *p* < .001, *d* = .62. Finally, negative gossip about the manager was significantly higher for perceived employee-directed managerial inaction (*M* = 3.88, *SD* = 1.57) than action (*M* = 2.38, *SD* = 1.29), *t*(159) = 6.56, *p* < .001, *d* = 1.04). H2a to H4a were supported.

To test our mediations, we constructed a dichotomous perceived employee-directed managerial inaction variable based on condition (perceived employee-directed managerial action condition = 0; perceived employee-directed managerial inaction condition = 1) and performed a bootstrapped mediation analysis using Mplus version 8.1 (10,000 resamples) to calculate confidence intervals for the indirect effects. Table 2 presents the parameter estimates and results of the mediation analysis. As predicted, perceived employee-directed managerial inaction had a significant negative indirect effect on manager-directed citizenship behavior (indirect effect = −1.23, *SE* = .22, 95% CI [−1.72, −.86]), a significant positive indirect effect on resistance behavior (indirect effect = .58, *SE* = .20, 95% CI [.23, 1.02]), and a significant positive indirect effect on negative gossip about the manager (indirect effect = .92, *SE* = .31, 95% CI [.37, 1.57]) through perceived manager trustworthiness. H2b to H4b were supported.

**Table 2.** Study 1: Parameter Estimates.

Path description	Coefficient	*SE*	95% CI
Perceived employee-directed managerial inaction → Perceived manager trustworthiness	−1.40***	.11	[−1.61, −1.18]
Perceived manager trustworthiness → Manager-directed citizenship behavior	.88***	.14	[.65, 1.19]
Perceived manager trustworthiness → Resistance behavior	−.41**	.13	[−.70, −.17]
Perceived manager trustworthiness → Negative gossip about the manager	−.66**	.21	[−1.11, −.27]
Perceived employee-directed managerial inaction → Manager-directed citizenship behavior	−.42	.22	[−.80, .06]
Perceived employee-directed managerial inaction → Resistance behavior	−.10	.22	[−.57, .29]
Perceived employee-directed managerial inaction → Negative gossip about the manager	.54	.32	[−.12, 1.15]

*Note. N* = 161*.* The perceived employee-directed managerial inaction IV contrasts the action condition (coded as 0) with the inaction condition (coded as 1). Coefficients represent unstandardized parameter estimates. Indirect effects are presented in the text.

*p < .05*.* **p < .01. ***p < .001.

#### Supplemental Analyses

Using the same analytic strategy as our main analyses, we also compared the perceived employee-directed managerial inaction and control conditions to provide further evidence that the significant effects are explained by perceived employee-directed managerial inaction. As Levene's test for the equality of variances was not significant, *F*(1, 160) = .024, *p* = .877, we performed a t-test in which equal variances were assumed. As expected, there were significant differences between the perceived employee-directed managerial inaction and control conditions for perceived manager trustworthiness (control condition *M* = 3.46, *SD* = .75; *t*(159) = 10.39, *p* < .001, *d* = 1.64), manager-directed citizenship behavior (control condition *M* = 3.38, *SD* = .70; *t*(159) = 9.11, *p* < 001, *d* = 1.44), resistance behavior (control condition *M* = 2.09, *SD* = .76; *t*(159) = 2.31, *p* = .022, *d* = .37), and negative workplace gossip (control condition *M* = 2.86, *SD* = 1.29; *t*(159) = 4.48, *p* < .001, *d* = .71). There were also significant indirect effects from perceived employee-directed managerial inaction (control condition = 0, managerial inaction condition = 1) to manager-directed citizenship behavior (indirect effect = −.89, *SE* = .15, 95% CI [−1.21, −.62]), resistance behavior (indirect effect = .39, *SE* = .14, 95% CI [.15, .69]), and negative workplace gossip (indirect effect = .76, *SE* = .22, 95% CI [.36, 1.23]) via perceived manager trustworthiness. All evidence was consistent with perceived employee-directed managerial inaction being a driving factor for the effects.

### Discussion

Study 1 provides initial evidence that perceived employee-directed managerial inaction has a negative effect on employees’ perceptions of manager trustworthiness and manager-directed behaviors. Consistent with our theoretical model, we found significant indirect effects of perceived employee-directed managerial inaction on employees’ manager-directed citizenship behavior, resistance behavior, and negative gossip about the manager via perceived manager trustworthiness. Moreover, the controlled experimental design of Study 1 helped to establish temporal ordering for perceived employee-directed managerial inaction and perceived manager trustworthiness as well as rule out alternative explanations, thereby enhancing internal validity. Overall, the findings from Study 1 were consistent with the argument that perceived employee-directed managerial inaction can impact employees’ generalized perceptions of their managers and have detrimental effects on manager outcomes.

## Scale Development and Validation

While Study 1 supported our theorized model using an experiment to prioritize internal validity, we deemed it necessary to examine perceived employee-directed managerial inaction with field surveys to provide evidence of external validity. To do so, we developed and validated a measure of perceived employee-directed managerial inaction using best practices (e.g., Colquitt et al., 2019; Cortina et al., 2020; Hinkin, 1998). Consistent with the unidimensional nature of our conceptualization, we chose a reflective measurement strategy that develops items with the aim of capturing the same underlying construct (i.e., perceived employee-directed managerial inaction) versus a formative measurement strategy that develops items with the aim of identifying distinct items that can be combined to capture a multi-dimensional construct (for a detailed discussion, see Edwards, 2011).

### Item Generation

Given that reflective measures are intended to have items that “describe the same underlying construct” and are therefore “conceptually interchangeable” (Edwards, 2011, p. 373), we generated an initial item pool of five items that closely reflected the conceptual definition of perceived employee-directed managerial inaction, then evaluated the items for clarity (see Podsakoff et al., 2016). We developed five items to be consistent with Hinkin's (1998) recommendation that most constructs can be appropriately assessed with four to six items. Items were worded to focus on employees’ *perceptions* related to a manager's perceived failure to engage in behaviors to avoid confounding the perceived employee-directed managerial inaction items with affective or cognitive responses.

### Content Validity

An item-sort task was used to test the substantive validity of the five items (i.e., the degree to which items reflect the intended construct versus other constructs; Holden & Jackson, 1979). Consistent with recommendations to perform this task with between 12–30 participants (see Anderson & Gerbing, 1991), we decided a priori to recruit 30 participants. Employed adults from the US and Canada were recruited from Prolific and paid £1 to participate (*N* = 30; 47% female; mean age = 31.77 years, *SD* = 6.86; mean organizational tenure = 5.25 years, *SD* = 4.94). Participants reported working in a range of industries, including business and financial operations (16.7%), computer and mathematics (16.7%), and education (10%).

To assess the substantive validity of the items, participants read five different (unnamed) construct definitions that describe managers’ responses to unpleasant or immoral situations, then sorted randomly presented items to the construct definition that each item best reflected (see Anderson & Gerbing, 1991). We included amoral management (Quade et al., 2022) to provide evidence that perceived employee-directed managerial inaction is an event-based phenomenon that is distinct from managers’ consistent lack of ethical guidance. Acquiescent silence (Van Dyne et al., 2003) was included to distinguish from passive disengagement. Informational justice (i.e., providing truthful and adequate explanations; Colquitt, 2001) and interpersonal justice (i.e., treating others with dignity and respect; Colquitt, 2001) were included to provide evidence that perceived employee-directed managerial inaction (an inactive response) is distinct from employees’ event-based evaluations of managers’ adherence to justice rules (an active response). After completing the item-sort task, participants rated the clarity of the perceived employee-directed managerial inaction items (1 = *hard to understand* to 7 = *very easy to understand*).

We retained four items that had strong definitional correspondence (mean p_sa_ = .79) and high item clarity ratings (*M* = 6.13, *SD* = 1.36). The retained items were: “(My manager) did not respond appropriately to prevent and/or address my unpleasant experience when they were expected to”; “did not engage in appropriate behaviors to prevent and/or address my unpleasant experience when they were expected to”; “failed to take appropriate actions to prevent and/or address my unpleasant experience when they were expected to”; and “failed to act to prevent and/or address my unpleasant experience when they were obligated to”. With evidence that the items were consistent with our conceptual definition and easy to understand, the items were deemed to be substantively valid.

### Convergent and Discriminant Validity

To establish convergent validity, we examined the newly developed scale with two theoretically related constructs. We predicted that perceived employee-directed managerial inaction should be negatively related to (1) *employees’ perceptions of organizational procedural justice* (4 items; Rupp & Cropanzano, 2002) because fair organizational procedures may provide guidance for how managers should act in certain circumstances and (2) *interdependence* (3 items; Pearce & Gregersen, 1991) because managers who have an interdependency with their employees are likely to refrain from behaviors that can undermine their relationship and/or employees’ performance.

To establish discriminant validity, we examined *employee trait negative affectivity* (7 items; Denollet, 2005), a dispositional tendency to experience negative emotions (Watson et al., 1988). Since perceived employee-directed managerial inaction reflects a lack of behavior in response to an event rather than a reaction to employees’ dispositions, we predicted perceived employee-directed managerial inaction is weakly or unrelated to trait negative affectivity.

We recruited employed adults via Prolific (final *N* = 192; 60% female; mean age = 37.50 years, *SD* = 9.62; mean organizational tenure = 5.38 years, *SD* = 5.09). Participants were from a range of industries, including education (19.8%), healthcare (15.1%), and government (11.5%). Perceived employee-directed managerial inaction was negatively related to organizational procedural justice (*r* = −.27, *p* < .001) and interdependence with the manager (*r* = −.38, *p* < .001), providing convergent validity evidence. Perceived employee-directed managerial inaction was not significantly related to trait negative affectivity (*r* = .05, *p* = .531), providing discriminant validity evidence.

#### Psychological Properties

Given that reflective measures are intended to have items that serve as alternative indicators of the same construct and therefore demonstrate “useful redundancy” (Edwards, 2011, p. 373), the reliability of a reflective measure should be relatively high with strong inter-item correlations. Consistent with these guidelines, the perceived employee-directed managerial inaction scale demonstrated excellent reliability (α = .98), high mean inter-item correlations (*r* = .92), and met Hu and Bentler's (1999) criteria for good model fit (*χ*^2^(2) = 15.935, *p* < .001, CFI = .989, SRMR = .006).^4^ Moreover, the perceived employee-directed managerial inaction scale also demonstrated distinctiveness, such that a 4-factor measurement model in which all variables were modeled as being distinct (i.e., perceived employee-directed managerial inaction, organizational procedural justice, interdependence, and trait negative affectivity) had an acceptable fit to the data, *χ*^2^(129) = 257.92, *p* < .001, CFI = .959, RMSEA = .072, SRMR = .040. The 4-factor model had significantly better fit than all alternative 3-factor models (smallest Δ*χ*^2^(3) = 262, *p* < .001). Moreover, the average variance explained (AVE) for the perceived employee-directed managerial inaction scale was .92, which is greater than the squared correlation between the constructs (largest squared correlation was .18), indicating that the scale was distinct from all other variables (see Fornell & Larcker, 1981). Overall, evidence indicated that the scale was a valid measure of perceived employee-directed managerial inaction and was deemed finalized. The Appendix provides the finalized scale.

## Study 2

While Study 1 supported our theorized model using an experiment to prioritize internal validity, we deemed it necessary to examine perceived employee-directed managerial inaction with surveys to provide evidence of external validity. Thus, Study 2 tests our full theoretical model (including employee psychological well-being which could not be tested with an experiment). We used a three-wave survey with employees who had pre-existing relationships with their managers. We also examine the incremental validity of perceived employee-directed managerial inaction beyond the constructs used to establish substantive validity.

### Sample and Procedure

Employed adults (United States, Canada, and the United Kingdom) were recruited via Prolific. Based on a power analysis for a mediation with weak correlations (*r* = .20), a 95% confidence interval, and a desired power of .80 (see Schoemann et al., 2017), we calculated a desired sample size of 314 participants. However, we recruited a larger sample to account for participant drop-out across the survey waves. We first ran a pre-screen survey to identify participants who had an aversive experience from someone other than their manager and their manager knew about this experience. A six-month window for a qualifying event was chosen since we were unsure of the base rate for perceived employee-directed managerial inaction and wanted to ensure a sufficient final sample size. Qualifying participants were invited to complete a three-wave survey that paid £5.32 (£2.28 for T1, £1.63 for T2, and £1.41 for T3). T1 assessed perceived employee-directed managerial inaction (predictor) along with demographics and our control variables. T2 assessed perceived manager trustworthiness (mediator). T3 assessed manager-directed citizenship behavior, resistance behavior, negative gossip about the manager, and employee psychological well-being (outcomes). We chose a two-week time interval between waves to reduce common method bias (see Podsakoff et al., 2012).

A total of 503 participants finished the survey and qualified for participation. Data from 3 participants was excluded for failing to meet an a priori cutoff of three (out of five) correctly answered attention checks. Of the 500 qualifying participants invited for T2, 457 completed the survey. However, data from 94 participants was excluded because participants reported changing jobs, no longer regularly interacting with the specified manager, or could not remember the event from the T1 survey. The remaining participants all met the cut-off of two out of three correctly answered attention checks. Of the 363 participants invited for T3, 349 completed the survey. However, data from 22 participants was excluded because participants changed jobs or reported no longer interacting with the manager, and data from 3 participants was excluded for failing to meet the a priori cut-off of two (out of three) correctly answered attention checks in the T3 survey. Finally, data from 6 participants was excluded due to the presence of univariate outliers (> 3 *SD*s). The retention rate between the Time 1 and Time 3 samples was 63%.

We explored potential attrition effects by comparing the participants retained in the final sample to the 57 participants who were invited to the subsequent surveys but did not respond. Independent samples t-tests indicated that those in the final sample were significantly older (*M* = 34.91, *SD* = 9.35) than those who did not respond (*M* = 30.09, *SD* = 9.33, *t*(372) = 3.21, *p* < .001). There were no significant differences for other demographics. Results were substantively similar with and without participant age controlled. Therefore, we report the results without this control variable (see Becker et al., 2016).

The final sample included 318 participants (75.2% female; mean age = 34.97 years, *SD* = 9.38; mean organizational tenure = 5.01 years, *SD* = 4.35; mean time reporting to their manager = 3.05 years, *SD* = 2.87; 88.1% identified as Caucasian, 4.4% as Asian, 2.2% as Black or African American). Participants were employed in a range of industries, with the most frequent being healthcare (19.2%), education (18.9%), and sales (7.9%).

### Measures

*Perceived employee-directed managerial inaction* was measured using the finalized scale. During the prescreen, participants were asked to recall and describe an experience of workplace harm (“Please recall an event that happened in the past six months in which someone other than your manager harmed you while you were at work (e.g., emotional, verbal, or physical harm) and your manager knew about this experience.”) and then respond to the scale items (1 = *not at all accurate* to 7 = *extremely accurate*). The question stem was: “The following questions are about your manager. For each statement below, indicate the extent to which your manager engaged in appropriate managerial behaviors to deal with the event and/or harm you described.”

*Psychological well-being* was assessed with Goldberg and William'’s (1988) 12-item scale (e.g., “(was) able to concentrate”; 1 = *never* to 4 = *always*).

The same scales and response scale formats from Study 1 were used to assess our behavioral outcomes. However, participants were asked to complete the measures for their actual managers and indicate the extent to which they engaged in behaviors over the past two weeks. Psychological well-being was also assessed over the past two weeks.

To ensure that perceived employee-directed managerial inaction explains incremental variance beyond existing conceptually similar constructs, we measured the four variables used to establish substantive validity (see scale development): informational justice (5-item scale; Colquitt, 2001), interpersonal justice (4-item scale; Colquitt, 2001), amoral management (4-item scale; Quade et al., 2022), and acquiescent silence (5-item scale; Van Dyne et al., 2003).

### Results

Before testing our hypotheses, we examined a 6-factor measurement model (i.e., perceived employee-directed managerial inaction, perceived manager trustworthiness, manager-directed citizenship behavior, resistance behavior, negative gossip about the manager, and psychological well-being) using Mplus version 8.1. Consistent with Study 1, perceived manager trustworthiness was modeled with three indicators, corresponding to the three dimensions of trustworthiness (ability, benevolence, and integrity; Mayer & Davis, 1999). While the RMSEA and SRMR met Hu and Bentler's (1999) criteria for good model fit, the CFI did not, *χ*^2^(579) = 1200.62, *p* < .001, CFI = .927, RMSEA = .058, SRMR = .052. As such, we further examined the psychometric properties of each individual scale in the model to determine what may have impacted the model fit. Whereas the model fit for the newly developed perceived employee-directed managerial inaction scale was excellent (CFI = .992, SRMR = .005), two other scales did not meet the criteria for good fit (psychological well-being: CFI = .857, SRMR = .072; citizenship behavior: CFI = .917, SRMR = .043). Further analysis identified the negatively worded items from the psychological well-being scale and an item from the citizenship behavior scale (“I defended my manager's opinion or suggestion”) as problematic. When these items were removed, the threshold for good model fit was met (χ^2^(362) = 658.138, *p* < .001, CFI = .957, RMSEA = .051, SRMR = .047). The bootstrapping results remained substantively similar when the analyses were rerun with the modified scales. Since these scales have been extensively used in the literature and may be subject to future meta-analyses, we report the results with the original/full published scales for psychological well-being and citizenship behavior.

Before proceeding to our hypothesis testing, we also examined the distinctiveness of the variables (using the full psychological well-being and citizenship behavior scales). The 6-factor model had a significantly better fit than all possible 5-factor models in which two of the 6 factors were combined into a single factor, smallest Δ*χ*^2^(5) = 350.31, *p* < .001, supporting the distinctiveness of the variables.^5^

Table 3 shows descriptive statistics, reliabilities, and correlations. A linear regression analysis tested the effect of perceived employee-directed managerial inaction on perceived manager trustworthiness (H1) and our dependent variables (H2a to H5a). Perceived employee-directed managerial inaction was significantly negatively related to employees’ perceptions of manager trustworthiness (*b* = −.22, *SE* = .02, *p* < .001; H1). H1 was supported. As predicted, perceived employee-directed managerial inaction was significantly negatively related to manager-directed citizenship behavior (*b* = −.30, *SE* = .04, *p* < .001; H2a) and psychological well-being (*b* = −.04, *SE* = .01, *p* < .001; H5a), and significantly positively related to employees’ resistance behavior (*b* = .04, *SE* = .01, *p* = .008; H3a), and negative gossip about the manager (*b* = .24, *SE* = .04, *p* < .001; H4a). H2a to H5a were supported.

**Table 3.** Study 2: Means, Standard Deviations, Zero-Order Correlations, and Reliabilities.

Variables	*M*	*SD*	1	2	3	4	5	6	7	8	9	10
1. Perceived employee-directed managerial inaction	3.60	2.12	(.98)									
2. Perceived manager trustworthiness	3.66	.91	−.60	(.97)								
3. Manager-directed citizenship behavior	4.15	1.57	−.39	.64	(.92)							
4. Resistance behavior	1.50	.64	.15**	−.22	−.19	(.81)						
5. Negative gossip about the manager	2.21	1.43	.37	−.53	−.35	.49	(.93)					
6. Employee well-being	2.98	.58	−.22	.34	.23	−.29	−.34	(.90)				
7. Informational justice	3.38	1.05	−.60	.75	.58	−.19	−.45	.30	(.90)			
8. Interpersonal justice	3.93	1.18	−.57	.69	.50	−.21	−.44	.38	.78	(.90)		
9. Amoral management	2.46	1.03	.54	−.52	−.39	.21	.36	−.20	−.51	−.44	(.86)	
10. Silence	2.48	1.34	.35	−.52	−.33	.23	.36	−.26	−.46	−.45	.54	(.89)

*Note. N* = 318. Reliabilities are displayed in parentheses.

*p* < .001 for all correlations reported in this table unless indicated otherwise.

**p < .01.

We examined the indirect effects (H2b to H5b) with bootstrapped mediation analyses (10,000 iterations) to obtain 95% confidence intervals. Table 4 displays the parameter estimates. Results indicated that perceived employee-directed managerial inaction had significant indirect effects on manager-directed citizenship behavior (indirect effect = −.32, *SE* = .04, 95% CI [−.41, −.24]; H2b), resistance behavior (indirect effect = .03, *SE* = .01, 95% CI [.01, .05]; H3b), negative gossip about the manager (indirect effect = .21, *SE* = .03, 95% CI [.15, .28]; H4b), and employee psychological well-being (indirect effect = −.04, *SE* = .01, 95% CI [−.07, −.02]; H5b) through perceived manager trustworthiness. H2b to H5b were supported.

**Table 4.** Study 2: Parameter Estimates.

Path description	Coefficient	*SE*	95% CI
Perceived employee-directed managerial inaction → Perceived manager trustworthiness	−.22^***^	.02	[−.27, −.18]
Perceived manager trustworthiness → Manager-directed citizenship behavior	1.44***	.16	[1.15, 1.78]
Perceived manager trustworthiness → Resistance behavior	−.13*	.06	[−.24, −.03]
Perceived manager trustworthiness → Negative gossip about the manager	−.95***	.15	[−1.27, −.67]
Perceived manager trustworthiness → Psychological well-being	.20***	.05	[.11, .30]
Perceived employee-directed managerial inaction → Manager-directed citizenship behavior	.02	.05	[−.08, .12]
Perceived employee-directed managerial inaction → Resistance behavior	.01	.02	[−.03, .04]
Perceived employee-directed managerial inaction → Negative gossip about the manager	.03	.05	[−.06, .12]
Perceived employee-directed managerial inaction → Psychological well-being	.00	.01	[−.02, .03]

*Note. N* = 318. Coefficients represent unstandardized parameter estimates. Indirect effects are presented in the text.

*p < .05. **p < .01. ***p < .001.

#### Supplemental Analyses

We examined whether perceived employee-directed managerial inaction explains incremental variance beyond informational justice, interpersonal justice, amoral management, and acquiescent silence. Following best practice guidance for the use of control variables (see Spector, 2021), we reran the analyses with the individual control variables. Results remained substantively similar except for the indirect effect of perceived employee-directed managerial inaction on resistance behavior via trustworthiness, which became non-significant when controlling for informational justice (indirect effect = .01, *SE* = .01, 95% CI [−.01, .02]), interpersonal justice (indirect effect = .01, *SE* = .01, 95% CI [−.01, .02]), amoral management (indirect effect = .01, *SE* = .01, 95% CI [−.003, .03]), and acquiescent silence (indirect effect = .012, *SE* = .011, 95% CI [−.01, .03]). However, these weaker effects for resistance behavior may be due to some items in the resistance scale (e.g., “I refused to carry out the request from my manager”) only being relevant if the manager has already made a request.

### Discussion

Study 2 demonstrated that perceived employee-directed managerial inaction can detract from perceived manager trustworthiness, which can have detrimental implications for managers and employees. By using a heterogeneous sample of employees in pre-existing relationships, Study 2 provides further evidence for external validity. Moreover, Study 2 demonstrated that perceived employee-directed managerial inaction explains unique variance beyond conceptually related variables, which provides further evidence for the distinctiveness and importance of perceived employee-directed managerial inaction.

## Study 3

Using a three-wave survey, Study 3 examines the replicability of the relationships between perceived employee-directed managerial inaction with our behavioral outcomes and psychological well-being. We also examine the incremental validity of perceived employee-directed managerial inaction (an event-based perception) beyond theoretically relevant leadership constructs (i.e., generalized perceptions of the manager's ongoing behavior across situations and time). We also recruited participants who perceived an incident of employee-directed managerial inaction within the past 14 days (versus the 6-month window used in Study 2) to capture behavioral reactions closer to the precipitating event, thereby enhancing internal validity. We also reduced the time separation between survey waves from two weeks to one week to reduce the potential for retrospective biases and alternative explanations.

To demonstrate the incremental validity of perceived employee-directed managerial inaction beyond existing leadership constructs, we examined passive and moral leadership styles, including laissez-faire leadership (i.e., the general absence of leadership and/or the avoidance of intervention; Bass & Avolio, 1990), management by exception—passive (i.e., a general style of leadership whereby a manager typically remains passive until problems become serious; Bass & Avolio, 1990), and transformational leadership (i.e., leadership style where a manager uses charisma, inspiration, intellectual stimulation, or individualized consideration; Bass, 1999). We also examined leader-member exchange (LMX; i.e., the quality of leader-member relationship that reflects the extent to which the two parties exchange resources, support, and loyalty beyond the formal contract; Liden et al., 2006) to rule out the possibility that the effect of perceived employee-directed managerial inaction is influenced by relationship quality with the manager.

### Sample and Procedure

Full-time employees from the United States, Canada, and the United Kingdom were recruited via Prolific. Using a prescreen survey, we identified participants that had experienced an aversive event (that their manager did not cause but was aware of) within the past 14 days. A total of 542 participants completed the T1 survey and qualified for participation. Data from 43 participants was excluded because they did not meet our inclusion criteria (e.g., could not recall the experience they reported at pre-screen or requested their data be deleted). Of the 499 qualifying participants invited for T2, 448 completed the survey. We excluded data from 12 who reported changing jobs or no longer regularly interacting with the specified manager and 1 whose age and gender did not match across T1 and T2. All remaining participants met the cut-off of three out of five correctly answered attention checks. Of the 435 participants invited for T3, 406 completed the survey. We excluded data from 6 participants who reported no longer interacting with the manager or requested their data be deleted. Data from 4 was excluded for failing to meet the a priori cut-off of two (out of three) correctly answered attention checks in the T3 survey. The retention rate between the Time 1 and Time 3 samples was 73.06%.

We explored potential attrition effects by comparing participants who were retained in the final sample to the 74 participants who were invited to the subsequent surveys but did not respond. Independent samples t-tests indicated that those in the final sample were older (*M* = 34.97, *SD* = 9.38) than those who did not respond (*M* = 30.09, *SD* = 9.17, *t*(468) = 1.94, *p* = .027). Those in the final sample also worked with their managers for longer (*M* = 3.44, *SD* = 4.02) than those who did not respond (*M* = 2.42, *SD* = 3.33, *t*(455) = 2.02, *p* = .044). There were no significant differences for other demographics. Results were substantively similar with and without participant age or tenure with their manager controlled. Therefore, we report the results without these control variables (see Becker et al., 2016).

The final sample included 396 participants (56.7% female; mean age = 37.38 years, *SD* = 9.88; mean organizational tenure = 6.13 years, *SD* = 6.21; mean time reporting to their manager = 3.44 years, *SD* = 4.02; 84.3% identified as Caucasian, 6.1% as Asian, 4.3% as Black or African American). Participants were employed in a range of industries, with the most frequent being education (15.9%), healthcare (15.7%), and sales (11.4%).

### Measures

The same scales from Study 2 were used but were assessed for the past week instead of the past two weeks. To examine the incremental validity of perceived employee-directed managerial inaction beyond theoretically relevant leadership constructs, we measured several leadership constructs using Bass and Avolio's (1990) Multifactor Leadership Questionnaire. More precisely, we measured *laissez-faire leadership* (4 items; e.g., “My manager avoids making decisions”), *management-by-exception*—*passive* (4 items: an example item is not provided for copyright reasons), both of which reflect passive leadership styles (e.g., perceptions of their leaders’ generalized tendencies that extend across events). We also measured *transformational leadership* (12 items; e.g., “My manager spends time teaching and coaching”) to distinguish perceived employee-directed managerial inaction from positive leadership styles that have been heavily studied. The response scale ranged from 1 = *not at all* to 5 = *frequently, if not always*. We also measured *LMX* using six items representing the affect and loyalty dimensions from Liden and Maslyn's (1998) multi-dimensional measure of LMX (e.g., “(In general) I like my manager very much as a person (affect)”; “My manager would come to my defense if I were attacked by others. (loyalty)”). The response scale ranged from 1 = *strongly disagree* to 5 = *strongly agree*. These dimensions were selected because they reflect the relational bond between an employee and their manager.

### Results

Before testing our hypotheses, we conducted a CFA using Mplus version 8.1 to examine a 6-factor measurement model (i.e., perceived employee-directed managerial inaction, perceived managerial trustworthiness, manager-directed citizenship behavior, resistance behavior, negative gossip about the manager, and psychological well-being). Consistent with the previous studies, perceived manager trustworthiness was modeled with three indicators, corresponding to the dimensions of trustworthiness (ability, benevolence, and integrity; Mayer & Davis, 1999). Similar to our previous studies, the CFI for the full measurement model fell below acceptable thresholds whereas the RMSEA and SRMR indicated good model fit, *χ*^2^(579) = 1586.46, *p* < .001, CFI = .912, RMSEA = .066, SRMR = .056. As such, we explored the individual scales to identify what scales may be detracting from model fit. Whereas the model fit for the newly developed perceived employee-directed managerial inaction scale was excellent (CFI = .99, SRMR = .006), the CFI for psychological well-being did not meet the criteria for acceptable fit (CFI = .839, SRMR = .072) and the same problematic item as identified in Study 2 detracted from the fit for citizenship behavior (CFI = .947, SRMR = .034). Similar to Study 2, when the problematic issues from the psychological well-being and citizenship scales were removed, the resulting measurement model met the threshold for acceptable fit across all indices, χ^2^(362) = 869.183, p < .001, CFI = .947, RMSEA = .059, SRMR = .046. Moreover, the bootstrapping results remained substantively similar when we reran the analyses with these modified scales. As such, we report the analyses using full published scales. We also examined the distinctiveness of the scales (using the full psychological well-being and citizenship behaviors scales). Results indicated that the 6-factor model had significantly better fit than all possible 5-factor models, smallest Δχ^2^(5) = 520.21, *p* < .001, which supported the distinctiveness of the variables.

Table 5 shows descriptive statistics, reliabilities, and correlations. A linear regression analysis indicated that perceived employee-directed managerial inaction was significantly negatively related to employees’ perceptions of manager trustworthiness (*b* = −.26, *SE* = .02, *p* < .001; H1). H1 was supported. Further, perceived employee-directed managerial inaction was significantly negatively related to manager-directed citizenship behavior (*b* = −.40, *SE* = .04, *p* < .001; H2a) and psychological well-being (*b* = −.07, *SE* = .01, *p* < .001; H5a), as well as significantly positively related to employees’ resistance behavior (*b* = .10, *SE* = .02, *p* < .001; H3a) and negative gossip about the manager (*b* = .29, *SE* = .03, *p* < .001; H4a). H2a to H5a were supported.

**Table 5.** Study 3: Means, Standard Deviations, Zero-Order Correlations, and Reliabilities.

Variables	*M*	*SD*	1	2	3	4	5	6	7	8	9	10
1. Perceived employee-directed managerial inaction	3.55	2.02	(.98)									
2. Perceived manager trustworthiness	3.76	.98	−.58	(.98)								
3. Manager-directed citizenship behavior	2.03	1.42	−.46	.61	(.92)							
4. Resistance behavior	1.51	.69	.34	−.32	−.22	(.85)						
5. Negative gossip about the manager	4.23	1.58	.41	−.48	−.30	.54	(.94)					
6. Employee psychological well-being	3.10	.59	−.27	.41	.30	−.33	−.32	(.91)				
7. Transformational leadership	3.26	.99	−.59	.80	.63	−.28	−.42	.40	(.96)			
8. Laissez-faire leadership	2.25	1.12	.63	−.66	−.48	.32	.42	−.37	−.69	(.91)		
9. Management by exception—Passive	2.49	1.19	.67	−.63	−.46	.30	.41	−.32	−.67	.82	(.92)	
10. LMX—Affect and loyalty	4.65	1.75	−.61	.80	.66	−.30	−.43	.40	.83	−.66	−.65	(.96)

*Note. N* = 396. Reliabilities are displayed in parentheses.

*p* < .001 for all correlations reported in this table.

Indirect effects (H2b to H5b) were examined using bootstrapped mediation analyses with 10,000 iterations using Mplus 8.1. Table 6 displays the parameter estimates from the model. Consistent with our prediction, perceived employee-directed managerial inaction had significant indirect effects on manager-directed citizenship behavior (indirect effect = −.28, *SE* = .04, 95% CI [−.35, −.21]; H2b), resistance behavior (indirect effect = .03, *SE* = .01, 95% CI [.01, .06]; H3b), negative gossip about the manager (indirect effect = .14, *SE* = .03, 95% CI [.08, .21]; H4b), and employee psychological well-being (indirect effect = −.06, *SE* = .01, 95% CI [−.09, −.04]; H5b) through perceived manager trustworthiness. H2b to H5b were supported.

**Table 6.** Study 3: Parameter Estimates.

Path description	Coefficient	*SE*	95% CI
Perceived employee-directed managerial inaction → Perceived manager trustworthiness	−.26^***^	.02	[−.30, −.21]
Perceived manager trustworthiness → Manager-directed citizenship behavior	1.07***	.12	[.83, 1.31]
Perceived manager trustworthiness → Resistance behavior	−.11*	.05	[−.21, −.03]
Perceived manager trustworthiness → Negative gossip about the manager	−.55***	.12	[−.80, −.33]
Perceived manager trustworthiness → Psychological well-being	.25***	.04	[.17, .33]
Perceived employee-directed managerial inaction → Manager-directed citizenship behavior	−.12*	.05	[−.22, −.03]
Perceived employee-directed managerial inaction → Resistance behavior	.07**	.02	[.03, .11]
Perceived employee-directed managerial inaction → Negative gossip about the manager	.15***	.04	[.07, .23]
Perceived employee-directed managerial inaction → Psychological well-being	−.01	.02	[−.04, .02]

*Note. N* = 396. Coefficients represent unstandardized parameter estimates. Indirect effects are presented in the text.

*p < .05. **p < .01. ***p < .001.

#### Supplemental Analyses

We also examined whether perceived employee-directed managerial inaction explains incremental variance beyond the leadership constructs. Results remained substantively similar when leadership variables were included, except for resistance behavior. The indirect effect of perceived employee-directed managerial inaction on resistance behavior via trustworthiness became non-significant when controlling for laissez-faire leadership (indirect effect = .03, *SE* = .03, 95% CI [−.003, .03]), management by exception—passive (indirect effect = .01, *SE* = .01, 95% CI [−.001, .03]), and LMX (indirect effect = .004, *SE* = .01, 95% CI [−.003, .02]). Similar to Study 2, these weaker effects for resistance behavior may be due to some resistance items being dependent on the manager first making a particular request.

### Discussion

Study 3 replicated our theorized relationships in pre-existing relationships with a one-week time separation between waves to reduce retrospective bias, thereby providing further evidence of internal and external validity. Study 3 also demonstrated the incremental validity of perceived employee-directed managerial inaction beyond relevant leadership constructs. This provides further evidence for the importance of examining how specific instances of managers’ inactive responses can impact employees’ perceptions of their managers’ trustworthiness and prompt behaviors to respond to the situation, even for leaders that generally engage in positive or negative behaviors across time and situations.

## General Discussion

By introducing perceived employee-directed managerial inaction and validating a measure, we provide the conceptual and methodological foundation to understand and examine the implications of an employee-directed inactive and undesirable event-based managerial response (see Cropanzano et al., 2017). Our findings reveal that perceived employee-directed managerial inaction is not benign but rather can have detrimental effects on employees’ psychological well-being and their behavioral reactions toward managers. We discuss the theoretical and practical significance of these findings below.

### Illuminating the Practical and Theoretical Importance of Managerial Inaction

By introducing perceived employee-directed managerial inaction, we answer calls to move beyond the emphasis on active behaviors to identify and understand constructs that reflect inactive responses (see Cropanzano et al., 2017; Folger & Cropanzano, 2001). Beyond introducing and providing the conceptual foundation for understanding perceived employee-directed managerial inaction, we also developed and validated a scale to further advance research related to this theoretically and practically important phenomenon. Conceptually, we provide support for the notion that the omission of action (inactive responses) is distinct from the commission of action. Further, although failing to act may be assumed to be benign because the manager did not *actively* harm the employee, our findings reinforce that perceived employee-directed managerial inaction can have detrimental implications. This supports the notion that managers’ responses do not have to be active to have detrimental effects while also demonstrating that perceived employee-directed managerial inaction is not aligned with ethical and/or responsible management practices (see Tsui, 2021).

Our findings also point to the importance of further understanding how different parties may perceive, experience, and/or be impacted by managerial inaction. We focused on *employees’* perceptions of managerial inaction. However, we recognize that managers and third parties (those that may observe or hear about the interaction but are not directly involved; Skarlicki & Kulik, 2004) may have differing perceptions or experiences of the same events. Indeed, managers, employees, and third parties may have access to different information related to the situation and/or weigh available information differently depending on their perspective (e.g., Bashshur et al., 2023). For example, when considering what may have prompted the manager to engage in employee-directed inaction, managers may be more likely to emphasize situational factors, whereas employees may be more likely to emphasize dispositional factors. This suggests that it is critical to explore how different parties may perceive, experience, and even conceptualize managerial inaction. For example, examining why managers may engage in inaction, how managerial inaction shapes the ethical standards in the workplace, and how to curtail managerial inaction may enhance practical guidance related to preventing and managing managerial inaction (see Antonakis, 2017; Tsui, 2021). This also suggests the importance of exploring how differing perspectives may create perceptual predicaments and conflicts (e.g., Bashshur et al., 2023; Bies, 1987) between parties that may need to be negotiated and resolved.

We focused on perceived employee-directed managerial inaction. However, this is likely to be a subset of a broader managerial inaction construct. We encourage scholars to develop constructs that capture managerial inaction related to other issues and/or targets. Identifying additional inactive constructs that reflect (un)desirable responses can also enhance understanding of managers’ and employees’ experiences within the workplace (see Cropanzano et al., 2017).

### The Impact of Perceived Employee-Directed Managerial Inaction on Generalized Perceptions of Managers

Within the workplace, perceived managerial trustworthiness provides information to employees about how they can navigate their relationship with the manager and is essential for promoting cooperation. While perceived managerial trustworthiness can be a relatively stable and enduring evaluation (e.g., Mayer et al., 1995), our findings demonstrate that perceived employee-directed managerial inaction can detract from perceived managerial trustworthiness. This is consistent with emerging event-based research indicating that a *single* discrete event can shift employees’ generalized perceptions (e.g., Kiefer et al., 2022) but also advances the event-based literature by demonstrating that *inactive* event-based responses can impact employees’ generalized evaluations. Moreover, our findings showcase that a *single* instance of perceived employee-directed managerial inaction may have negative long-term implications for how employees perceive and navigate their relationship with the manager. Importantly, these effects emerged regardless of managers’ typical leadership styles and even for those managers that have a good quality relationship with their employees (high LMX). Together, these findings reinforce the importance of effectively managing everyday events since these can impact generalized perceptions—and doing so is critical for all managers, regardless of their typical leadership style.

Previous research has indicated that undesirable events can be impactful and difficult to overcome. While some scholars have argued that positive events can “undo” the effects of negative events (Fredrickson et al., 2000), others have argued that this can be difficult to achieve and may require multiple positive events to overcome a single negative event (e.g., Baumeister et al., 2001). Although it is possible that engaging in active and desirable responses in the aftermath of perceived employee-directed managerial inaction may mitigate the negative effects of these experiences, it is also possible that perceived employee-directed managerial inaction may serve as an “anchoring” event. More precisely, anchoring events occur when there is an event in which the actions of one party positively or negatively differs from “expectations given the decision rules applied to the relationships” and the other party is highly dependent on the party that deviated from expectations (Ballinger & Rockmann, 2010, p. 376). Given that perceived employee-directed managerial inaction occurs within a manager-employee relationship that is characterized by dependency and involves the perception that the manager failed to enact their managerial duties, perceived employee-directed managerial inaction may meet the criteria for an anchoring event. This is important because an anchoring event can “suddenly and durably” shift relationships (Ballinger & Rockmann, 2010, p. 373), including what employees may expect from their managers (e.g., the perceived rules that govern the relationship). This suggests that exploring the long-term impact of perceived employee-directed managerial inaction is critical not only to understand whether this fundamentally shifts the relationship but also whether managers may compensate for these events and/or redeem themselves (e.g., Bies et al., 2021).

### Understanding the Impact of Perceived Employee-Directed Managerial Inaction

Our findings support the theoretical argument that perceived employee-directed managerial inaction can prompt employees’ self-protective responses, directly and via perceived managerial trustworthiness. Beyond recognizing that employees can negatively react to the manager, it is also important to consider how these negative reactions may also (un)intentionally undermine managers’ effectiveness. For example, managers often rely on citizenship behaviors to receive support from their employees (Organ, 1988), whereas minimizing resistance behaviors is important for avoiding disruptions to workflows and enabling managers to advance their objectives (Tepper et al., 1998). Further, while employees may engage in negative gossip about the manager to protect against the risk of potential exploitation, this may have detrimental effects for the manager (e.g., Brady et al., 2017).

Following target similarity models (e.g., Lavelle et al., 2007), we emphasized employees’ direct responses toward their managers (e.g., manager-directed citizenship behaviors, manager-directed resistance behaviors, negative gossip about the manager). However, we also examined the impact of perceived employee-directed managerial inaction on employee well-being because it is a theoretically relevant and impactful outcome for employees. Moreover, employee well-being can also have broader implications for employee and organizational functioning (e.g., impacting performance and turnover; Wright & Bonett, 2007; Wright & Cropanzano, 2000). Our findings supported the notion that perceived employee-directed managerial inaction can diminish employee well-being, indicating that these experiences are also negative for employees and may also have broader implications for employee effectiveness. However, perceived employee-directed managerial inaction may also impact employees in other personally relevant ways in the short-term (e.g., eliciting negative emotions) and long-term (e.g., detracting from job satisfaction). As such, further exploring how perceived employee-directed managerial inaction may impact employee, manager, team, and organizational functioning over time is critical.

### Expanding on an Event-Based Approach: From Single to Multiple Events

While our findings shed light on the processes that can emerge in the wake of a single instance of perceived employee-directed managerial inaction, it is also critical to explore how employees react to multiple events. Indeed, previous research has demonstrated that frequent negative events (e.g., Kiefer et al., 2025) and/or inconsistency between positive and negative events (e.g., Matta et al., 2017) may be especially impactful or distressing for employees. Accordingly, future research should explore how previous instances of action or inaction can impact employees’ reactions to a current instance of perceived employee-directed managerial inaction, how the combination of inaction and action events may be experienced (including how perceived managerial inaction directed at other sources, such as customers, may impact employees’ experiences), as well as how multiple events can aggregate to impact downstream reactions including employees’ expectations for their manager and organization (e.g., Kiefer et al., 2022, 2025). Similarly, the social context surrounding inaction may also be a fruitful avenue for future research. This may include how others’ experiences with the same manager may impact one's own interpretations of the event (e.g., Hillebrandt & Barclay, 2017) and/or whether employees are likely to engage in helping behaviors towards their colleagues to manage their reactions to the situation and receive support (e.g., Barclay & Kiefer, 2014). For example, it is possible that employees may connect with and/or receive support from their colleagues (e.g., through peer citizenship behaviors) to compensate for the harmful effects of the manager, which may expand the (positive or negative) implications of managerial inaction beyond managers and employees to include coworker and/or team functioning.^6^

### Practical Implications

Our studies highlight several important practical implications for managers and organizations. First, by detracting from perceptions of trustworthiness, perceived employee-directed managerial inaction is not benign but can have negative implications for managers and the manager-employee relationship. Further, these detrimental effects may not be limited to the employees who experience perceived employee-directed managerial inaction directly but may also include others who hear about the inaction from others (e.g., via negative gossip, which may also detract from employees’ perceptions of the managers’ reputation; Jones & Skarlicki, 2005). This suggests that a *single* instance of perceived employee-directed managerial inaction can have significant detrimental implications for managers.

Second, the negative implications of perceived employee-directed managerial inaction also extend to employees’ psychological well-being. While this can have damaging effects for employees, employee well-being has also been linked to a range of outcomes that are important for organizations, including employee performance (e.g., Wright & Cropanzano, 2000) and turnover (e.g., Wright & Bonett, 2007). Thus, managers should avoid engaging in employee-directed managerial inaction not only because of its detrimental effects for themselves but also for their employees and organizations.

Third, organizations should increase managers’ awareness of the potential negative consequences associated with employee-directed managerial inaction. This may be especially important given experimental evidence from social psychology indicating that people tend to hold an “omission bias” in which they assume that actively harming others is more morally wrong than failing to act (Yeung et al., 2022). Organizations may also benefit from including information about employee-directed managerial inaction in their training programs. This is important not only for preventing negative consequences for employees and managers but also because managers are typically perceived as representatives of organizations (Shoss et al., 2013). As such, employees may also hold organizations accountable for perceived employee-directed managerial inaction. This concern is supported by the flurry of lawsuits and media articles outlining the negative impact of perceived employee-directed managerial inaction on companies and how employees are attempting to hold organizations responsible for their managers’ perceived inaction. Thus, it is imperative for organizations to take steps to prevent and mitigate employee-directed managerial inaction and its consequences.

### Strengths, Limitations, and Future Directions

We developed and validated our perceived employee-directed managerial inaction scale using a rigorous multi-study process that followed best practice recommendations. The scale demonstrated strong psychometric properties and empirical evidence supported substantive, convergent, and discriminant validity. Although our intention was to capture inactive/undesirable behaviors, we acknowledge the possibility that some participants may interpret the items in ways that may align with active/undesirable behaviors. For example, a high score on the item “did not engage in appropriate behaviors to prevent and/or address my unpleasant experience” could reflect that the manager did not act when expected or that the manager acted in a way the employee did not deem appropriate. To provide reassurance that our scale is capturing what was intended and aligned with our conceptualization, we further analyzed the scale items. We used the item “(My manager) failed to act to prevent and/or address my unpleasant experience when they were obligated to”) as a comparison since it has strong face validity for inactive/undesirable behaviors. Results indicated that this item had substantively similar psychometric properties to the other items in the scale and the results across multiple studies indicate that our scale is unidimensional, which provides confidence that our operationalization reflects the *absence of appropriate action* rather than the *presence of inappropriate action*. However, we recognize that strong evidence for the validity emerges over time as the scale is used in further research (see Bennett & Robinson, 2000). Therefore, we encourage future research to further explore how employees may perceive managers’ responses (e.g., what is perceived as inaction versus action as well as appropriate versus inappropriate) and the potential differential effects of these assessments.

We used a multi-method approach to test our theoretical model that included an experiment and two multi-wave surveys, all of which used employee samples. Self-report measures were used because employees are in the best position to report on their perceptions as well as negative behaviors (see Berry et al., 2012). Following Podsakoff et al. (2012), we used strategies to minimize common method bias (e.g., assessing scales following temporal ordering; separating our predictor, mediator, and outcomes across study waves). To enhance internal validity, the experiment manipulated perceived employee-directed managerial inaction and used random assignment to rule out alternative explanations. Using an experimental methodology was also important to provide confidence in the causal effects of perceived employee-directed managerial inaction on perceived managerial trustworthiness (e.g., to reduce potential concerns about endogentity; see Antonakis et al., 2010). To provide evidence of ecological validity, we complemented the experiment with two multi-wave surveys using real-life instances of perceived employee-directed managerial inaction. To enhance generalizability, our multi-wave surveys used two (Study 2) or one-week (Study 3) separations between waves. Whereas Study 2 examined instances of perceived employee-directed managerial inaction from the last six months, Study 3 focused on the last 14 days to reduce retrospective biases and enhance internal validity. We also followed best practices from Antonakis et al. (2010) to reduce potential concerns about endogeneity in survey studies (e.g., measuring theoretically relevant control variables, following best practices to reduce common method bias). Our results replicated across methodologies, thereby enhancing confidence in the findings. Nonetheless, we encourage future research to provide further confidence in the causal ordering of the variables and rule out potential endogeneity concerns.

While this research demonstrated the importance of perceived employee-directed managerial inaction for employee and manager outcomes, future research may benefit from exploring boundary conditions. For example, previous research has demonstrated that prior trust/commitment may attenuate (Robinson, 1996) or exacerbate (Brockner et al., 1992; Skarlicki et al., 2008) employees’ reactions to negative events. This suggests the importance of exploring how employees’ pre-existing perceptions may impact their experiences of employee-directed managerial inaction. Similarly, it may be helpful to explore how employees’ attributions and/or perceptions of the manager's motives for employee-directed managerial inaction impact employees’ reactions to these events (e.g., Matta et al., 2020), including whether employees have confidence in their manager's capacity or willingness to intervene for future events.

Finally, we focused on employees’ perceptions to understand their experiences and reactions to employee-directed managerial inaction. However, Fischer et al. (2023) argued that it is also critical to pinpoint which manager/leader behaviors may be especially likely impact employees. This suggests that it is not only important to explore *what* behaviors may be perceived as managerial inaction but also *when* and *why* these behaviors may be interpreted this way. Exploring perceptual differences between managers and employees may also provide fruitful insights. For instance, employees and managers may have perceptual differences related to whether the manager was aware of the experience, expected to act, that there was a failure to act, or that it was inappropriate to withhold an action (e.g., the manager may have responded in ways that the employee was not aware and/or perceived that acting would result in worse outcomes that failing to act). This suggests that future research should also (a) adopt an actor-centric approach (e.g., Zhong & Robinson, 2021) to shed light on *why* managerial inaction occurs from the manager perspective, (b) consider what gives rise to perceptual differences (e.g., differences in perceived awareness, intent, and/or behavioral execution), and (c) how these perceptual differences can be identified and effectively navigated (e.g., Bashshur et al., 2025; Bies, 1987; Shaw et al., 2003). These insights are likely to be especially important for enhancing leader development and interventions related to managing managerial inaction.

## Conclusion

By introducing perceived employee-directed managerial inaction, we answered scholarly calls to (a) identify constructs that reflect inactive and undesirable responses (e.g., Cropanzano et al., 2017), (b) examine the influence of event-based responses on generalized perceptions (e.g., Kiefer et al., 2022), and (c) promote responsible management practices as well as reduce the research-practice gap (e.g., Tsui, 2021). Our findings showed that perceived employee-directed managerial inaction has negative implications for how employees perceive and respond to their manager as well as detract from employee well-being. Taken together, this suggests that *in*active manager behaviors can have detrimental effects on both employees *and* managers. Thus, our findings highlight the theoretical and practical significance of recognizing that perceived employee-directed managerial inaction is not benign but rather can have significant detrimental implications that are not aligned with responsible management practices. We encourage scholars to continue to investigate perceived employee-directed managerial inaction while also broadening to explore other forms of managerial inaction and their impact within the workplace.

## Notes

1. We focus on introducing an inactive and undesirable construct. However, we encourage scholars to also consider filling the quadrant that reflects inactive and desirable constructs.

2. Given that perceived employee-directed managerial inaction is conceptually defined as occurring in response to an aversive (e.g., harmful or unpleasant) employee experience, our manipulation included a statement across all conditions that referred to “bad behavior” from a coworker to align with this conceptualization. To assess participants’ ratings of the coworker's behavior, we also asked participants to indicate on a 5-point Likert scale (ranging from 1 = *not at all bad* to 5 = *extremely bad*) “How bad was the coworker's behavior?” after the main study measures were completed. Results indicated that participants perceived the coworker's behavior to be “bad”, with a mean that was higher than the midpoint on the scale (*M* = 4.37; *SD* = .797). Moreover, there were no significant differences for these ratings across conditions, *F*(2, 233) = .625, *p* = .536, indicating that our effects were unlikely to be due to differences related to perceptions of the coworker's behavior.

3. Across all studies, we also examined whether loading all the trustworthiness items on one factor fit the data better. Empirical evidence indicated that modeling trustworthiness as having three indicators (ability, benevolence, and integrity) that predict a latent perceived managerial trustworthiness factor had a significantly better fit to the data. As such, we use this approach for all studies.

4. Given that the items in reflective measures are intended to be conceptually interchangeable (see Edwards, 2011), we also examined whether the scale performed differently when shortened versions were used (e.g., one, two, or three items). Empirical evidence indicated that the shortened scales continued to reflect a unidimensional structure, reliability (computed for two and three item versions) remained high (ranging from .95 to .975), and the results were substantively similar regardless of the number or combination of items used. This provides further evidence that the scale development and validation process aligned with the goals of a reflective measure. Further, this suggests that scholars who would like to use single items from the scale (e.g., for diary studies) can select the item that they believe best aligns with their research question or intended purpose since the items behave similarly.

5. In Studies 2 and 3, we also conducted CFAs to further examine the distinctiveness of the variables based on time of measurement (i.e., which survey wave the variable was measured in) and whether the construct is perceptual (i.e., perceived employee-directed managerial inaction, perceived managerial trustworthiness, and employee-well-being) or behavioral (i.e., manager-directed citizenship behavior, resistance behavior, and negative gossip about the manager). Our measurement model had a significantly better fit than these alternative models (Time of measurement: Δχ^2^(12) = 2658.20 for Study 2; Δχ^2^(12) = 3546.66 for Study 3; perceptual and behavioral constructs: Δχ^2^(14) = 3528.70 for Study 2; Δχ^2^(14) = 4756.03 for Study 3), indicating sufficient distinctiveness.

6. We thank an anonymous reviewer for this point.

## Appendix

### Perceived Employee-Directed Managerial Inaction Scale

The following questions are about your manager. For each statement below, indicate the extent to which your manager engaged in appropriate managerial behaviors to deal with the event and/or harm you described (1 = *not at all accurate* to 7 = *extremely accurate*).

My manager…

1. Did not respond appropriately to prevent and/or address my unpleasant experience when they were expected to
2. Did not engage in appropriate behaviors to prevent and/or address my unpleasant experience when they were expected to
3. Failed to take appropriate actions to prevent and/or address my unpleasant experience when they were expected to
4. Failed to act to prevent and/or address my unpleasant experience when they were obligated to

## References

[bibr1-15480518251352592] Aguinis H. Bradley K. J. (2014). Best practice recommendations for designing and implementing experimental vignette methodology studies. Organizational Research Methods, 17(4), 351-371. 10.1177/1094428114547952

[bibr2-15480518251352592] Anderson J. C. Gerbing D. W. (1991). Predicting the performance of measures in a confirmatory factor analysis with a pretest assessment of their substantive validities. Journal of Applied Psychology, 76(5), 732-740. 10.1037/0021-9010.76.5.732

[bibr3-15480518251352592] Anderson M. H. Sun P. Y. (2017). Reviewing leadership styles: Overlaps and the need for a new ‘full-range’ theory. International Journal of Management Reviews, 19(1), 76-96. 10.1111/ijmr.12082

[bibr4-15480518251352592] Antonakis J. (2017). On doing better science: From thrill of discovery to policy implications. The Leadership Quarterly, 28(1), 5-21. 10.1016/j.leaqua.2017.01.006

[bibr5-15480518251352592] Antonakis J. Bendahan S. Jacquart P. Lalive R. (2010). On making causal claims: A review and recommendations. The Leadership Quarterly, 21(6), 1086-1120. 10.1016/j.leaqua.2010.10.010

[bibr6-15480518251352592] Ballinger G. A. Rockmann K. W. (2010). Chutes versus ladders: Anchoring events and a punctuated-equilibrium perspective on social exchange relationships. Academy of Management Review, 35(3), 373-391. 10.5465/amr.35.3.zok373

[bibr7-15480518251352592] Barclay L. J. Kiefer T. (2014). Approach or avoid? Exploring overall justice and the differential effects of positive and negative emotions. Journal of Management, 40(7), 1857-1898. 10.1177/0149206312441833

[bibr8-15480518251352592] Bashshur M. R. Barclay L. J. Fortin M. (2025). Of headlamps and marbles: A motivated perceptual approach to the dynamic and dialectic nature of fairness. Organizational Psychology Review, *15*(2), 127–155. 10.1177/20413866231199068 PMC12157670 40511374

[bibr9-15480518251352592] Bass B. M. (1999). Two decades of research and development in transformational leadership. European Journal of Work and Organizational Psychology, 8(1), 9-32. 10.1080/135943299398410

[bibr10-15480518251352592] Bass B. M. Avolio B. J. (1990). Transformational leadership development: Manual for the Multifactor Leadership Questionnaire. *Consulting Psychologists Press* .

[bibr11-15480518251352592] Baumeister R. F. Bratslavsky E. Finkenauer C. Vohs K. D. (2001). Bad is stronger than good. Review of General Psychology, 5(4), 323-370. 10.1037/1089-2680.5.4.323

[bibr12-15480518251352592] Becker T. E. Atinc G. Breaugh J. A. Carlson K. D. Edwards J. R. Spector P. E. (2016). Statistical control in correlational studies: 10 essential recommendations for organizational researchers. Journal of Organizational Behavior, 37(2), 157-167. 10.1002/job.2053

[bibr13-15480518251352592] Bennett R. J. Robinson S. L. (2000). Development of a measure of workplace deviance. Journal of Applied Psychology, 85(3), 349-360. 10.1037/0021-9010.85.3.349 10900810

[bibr14-15480518251352592] Berry C. M. Carpenter N. C. Barratt C. L. (2012). Do other-reports of counterproductive work behavior provide an incremental contribution over self-reports? A meta-analytic comparison. Journal of Applied Psychology, 97(3), 613-636. 10.1037/a0026739 22201245

[bibr15-15480518251352592] Bies R. J. (1987). The predicament of injustice: The management of moral outrage. Research in Organizational Behavior, 9, 289-319.

[bibr16-15480518251352592] Bies R. J. Tripp T. M. Barclay L. J. (2021). Second acts and second chances: The bumpy road to redemption. Journal of Management Inquiry, 30(4), 371-384. 10.1177/105649262098685

[bibr17-15480518251352592] Brady D. L. Brown D. J. Liang L. H. (2017). Moving beyond assumptions of deviance: The reconceptualization and measurement of workplace gossip. Journal of Applied Psychology, 102(1), 1-25. httpsss://doi.org/http://doi.org/10.1037/apl0000164 https://doi.org/10.1037/apl0000164 27732002 10.1037/apl0000164

[bibr18-15480518251352592] Brockner J. Tyler T. R. Cooper-Schneider R. (1992). The influence of prior commitment to an institution on reactions to perceived unfairness: The higher they are, the harder they fall. Administrative Science Quarterly, 37(2), 241-261. 10.2307/2393223

[bibr19-15480518251352592] Brown M. E. Treviño L. K. Harrison D. A. (2005). Ethical leadership: A social learning perspective for construct development and testing. Organizational Behavior and Human Decision Processes, 97(2), 117-134. 10.1016/j.obhdp.2005.03.002

[bibr20-15480518251352592] Chughtai A. Byrne M. Flood B. (2015). Linking ethical leadership to employee well-being: The role of trust in supervisor. Journal of Business Ethics, 128(3), 653-663. 10.1007/s10551-014-2126-7

[bibr21-15480518251352592] Colquitt J. A. (2001). On the dimensionality of organizational justice: A construct validation of a measure. Journal of Applied Psychology, 86(3), 386-400. 10.1037/0021-9010.86.3.386 11419799

[bibr22-15480518251352592] Colquitt J. A. Sabey T. B. Rodell J. B. Hill E. T. (2019). Content validation guidelines: Evaluation criteria for definitional correspondence and definitional distinctiveness. Journal of Applied Psychology, 104(10), 1243-1265. 10.1037/apl0000406 30945879

[bibr23-15480518251352592] Colquitt J. A. Scott B. A. LePine J. A. (2007). Trust, trustworthiness, and trust propensity: A meta-analytic test of their unique relationships with risk taking and job performance. Journal of Applied Psychology, 92(4), 909-927. 10.1037/0021-9010.92.4.909 17638454

[bibr24-15480518251352592] Cortina J. M. Sheng Z. Keener S. K. Keeler K. R. Grubb L. K. Schmitt N. Tonidandel S. Summerville K. M. Heggestad E. D. Banks G. C. (2020). From alpha to omega and beyond! A look at the past, present, and (possible) future of psychometric soundness in the Journal of Applied Psychology. Journal of Applied Psychology, 105(12), 1351-1381. 10.1037/apl0000815 32772525

[bibr25-15480518251352592] Cropanzano R. Anthony E. L. Daniels S. R. Hall A. V. (2017). Social exchange theory: A critical review with theoretical remedies. Academy of Management Annals, 11(1), 479-516. 10.5465/annals.2015.0099

[bibr26-15480518251352592] Cropanzano R. Byrne Z. S. Bobocel D. R. Rupp D. E. (2001). Moral virtues, fairness heuristics, social entities, and other denizens of organizational justice. Journal of Vocational Behavior, 58(2), 164-209. 10.1006/jvbe.2001.1791

[bibr27-15480518251352592] Dalal R. S. Lam H. Weiss H. M. Welch E. R. Hulin C. L. (2009). A within-person approach to work behavior and performance: Concurrent and lagged citizenship-counterproductivity associations, and dynamic relationships with affect and overall job performance. Academy of Management Journal, 52(5), 1051-1066. httpsss://doi.org/http://doi.org/10.5465/amj.2009.44636148 https://doi.org/10.5465/amj.2009.44636148

[bibr28-15480518251352592] Denollet J. (2005). DS14: Standard assessment of negative affectivity, social inhibition, and type D personality. Psychosomatic Medicine, 67(1), 89-97. 10.1097/01.psy.0000149256.81953.49 15673629

[bibr29-15480518251352592] Dirks K. T. Ferrin D. L. (2002). Trust in leadership: Meta-analytic findings and implications for research and practice. Journal of Applied Psychology, 87(4), 611-628. 10.1037/0021-9010.87.4.611 12184567

[bibr30-15480518251352592] Edwards J. R. (2011). The fallacy of formative measurement. Organizational Research Methods, 14(2), 370-388. 10.1177/1094428110378369

[bibr31-15480518251352592] Feinberg M. Willer R. Stellar J. Keltner D. (2012). The virtues of gossip: Reputational information sharing as prosocial behavior. Journal of Personality and Social Psychology, 102(5), 1015-1030. 10.1037/a0026650 22229458

[bibr32-15480518251352592] Fischer T. Hambrick D. C. Sajons G. B. Van Quaquebeke N. (2023). Leadership science beyond questionnaires. The Leadership Quarterly, 34(6), 101752. 10.1016/j.leaqua.2023.101752

[bibr33-15480518251352592] Folger R. Cropanzano R. (2001). Fairness theory: Justice as accountability. In Greenberg J. Cropanzano R. (Eds.), Advances in organization justice (pp. 1-55). Stanford University Press.

[bibr34-15480518251352592] Fornell C. Larcker D. F. (1981). Evaluating structural equation models with unobservable variables and measurement error. Journal of Marketing Research, 18(1), 39-50. 10.2307/3151312

[bibr35-15480518251352592] Fredrickson B. L. Mancuso R. A. Branigan C. Tugade M. M. (2000). The undoing effect of positive emotions. Motivation and Emotion, 24(4), 237-258. 10.1023/A:1010796329158 21731120 PMC3128334

[bibr36-15480518251352592] Goldammer P. Annen H. Stöckli P. L. Jonas K. (2020). Careless responding in questionnaire measures: Detection, impact, and remedies. The Leadership Quarterly, 31(4), 101384. 10.1016/j.leaqua.2020.101384

[bibr37-15480518251352592] Goldberg D. P. Williams P. (1988). A user’s guide to the general health questionnaire. NFER-Nelson.

[bibr38-15480518251352592] Good D. (2022, July 15). Hazing lawsuit alleges Brownsboro ISD baseball team had history of sexual harassment. *KETK & FOX51*, www.ketk.com/news/local-news/hazing-lawsuit-alleges-brownsboro-isd-baseball-team-had-history-of-sexual-harassment/.

[bibr39-15480518251352592] Greenberg J. Bies R. J. Eskew D. E. (1991). Establishing fairness in the eye of the beholder: Managing impressions of organizational justice. In Giacalone R. A. Rosenfeld P. (Eds.), Applied impression management: How image-making affects managerial decisions (pp. 111-132). Sage Publications, Inc.

[bibr40-15480518251352592] Hauser D. J. Ellsworth P. C. Gonzalez R. (2018). Are manipulation checks necessary? Frontiers in Psychology, 9, 998. 10.3389/fpsyg.2018.00998 29977213 PMC6022204

[bibr41-15480518251352592] Helliwell J. F. Huang H. (2011). Well-being and trust in the workplace. Journal of Happiness Studies, 12(5), 747-767. 10.1007/s10902-010-9225-7

[bibr42-15480518251352592] Hillebrandt A. Barclay L. J. (2017). Observing others’ anger and guilt can make you feel unfairly treated: The interpersonal effects of emotions on justice-related reactions. Social Justice Research, 30(3), 238-269. 10.1007/s11211-017-0290-5

[bibr43-15480518251352592] Hinkin T. R. (1998). A brief tutorial on the development of measures for use in survey questionnaires. Organizational Research Methods, 1(1), 104-121. 10.1177/109442819800100106

[bibr44-15480518251352592] Holden R. R. Jackson D. N. (1979). Item subtlety and face validity in personality assessment. Journal of Consulting and Clinical Psychology, 47(3), 459-468. 10.1037/0022-006X.47.3.459

[bibr45-15480518251352592] Hu L. T. Bentler P. M. (1999). Cutoff criteria for fit indexes in covariance structure analysis: Conventional criteria versus new alternatives. Structural Equation Modeling: A Multidisciplinary Journal, 6(1), 1-55. 10.1080/10705519909540118

[bibr46-15480518251352592] Iyengar R. (2021, August 3). The Activision Blizzard lawsuit could be a watershed moment for the business world. Here's why. *CNN Business*. https://www.cnn.com/2021/08/03/tech/activision-blizzard-employee-backlash-kotick/index.html.

[bibr47-15480518251352592] Jones D. A. Skarlicki D. P. (2005). The effects of overhearing peers discuss an authority’s fairness reputation on reactions to subsequent treatment. Journal of Applied Psychology, 90(2), 363-372. 10.1037/0021-9010.90.2.363 15769244

[bibr48-15480518251352592] Kahan M. (1989). Causation and incentives to take care under the negligence rule. The Journal of Legal Studies, 18(2), 427-447. 10.1086/468154

[bibr49-15480518251352592] Kiefer T. Barclay L. J. Conway N. (2025). Applying event system theory to organizational change: The importance of everyday positive and negative events. Journal of Management, *51*(3), 1066-1095. 10.1177/014920632412372

[bibr50-15480518251352592] Kiefer T. Barclay L. J. Conway N. Briner R. (2022). An event-based approach to psychological contracts: The importance of examining everyday broken and fulfilled promises as discrete events. Journal of Organizational Behavior, 43(8), 1377-1395. 10.1002/job.2656

[bibr51-15480518251352592] Lavelle J. J. Rupp D. E. Brockner J. (2007). Taking a multifoci approach to the study of justice, social exchange, and citizenship behavior: The target similarity model. Journal of Management, 33(6), 841-866. 10.1177/0149206307307635

[bibr52-15480518251352592] Liden R. C. Erdogan B. Wayne S. J. Sparrowe R. T. (2006). Leader-member exchange, differentiation, and task interdependence: Implications for individual and group performance. Journal of Organizational Behavior, 27(6), 723-746. 10.1002/job.409

[bibr53-15480518251352592] Liden R. C. Maslyn J. M. (1998). Multidimensionality of leader-member exchange: An empirical assessment through scale development. Journal of Management, 24(1), 43-72. 10.1016/S0149-2063(99)80053-1

[bibr54-15480518251352592] Lind E. A. (2001). Fairness heuristic theory: Justice judgments as pivotal cognitions in organizational relations. In Greenberg J. Cropanzano R. (Eds.), Advances in organizational justice (pp. 56-88). Stanford University Press.

[bibr55-15480518251352592] Lindquist K. A. Satpute A. B. Wager T. D. Weber J. Barrett L. F. (2016). The brain basis of positive and negative affect: Evidence from a meta-analysis of the human neuroimaging literature. Cerebral Cortex, 26(5), 1910-1922. 10.1093/cercor/bhv001 25631056 PMC4830281

[bibr56-15480518251352592] Liu D. Morgeson F. P. Zhu J. Fan X. (2023). Event-oriented organizational behavior research: A multilevel review and agenda for future research. Journal of Management, 49(6), 2148-2186. 10.1177/014920632311620

[bibr57-15480518251352592] Long C. P. (2016). Mapping the main roads to fairness: Examining the managerial context of fairness promotion. Journal of Business Ethics, 137(4), 757-783. 10.1007/s10551-015-2749-3

[bibr58-15480518251352592] Matta F. K. Sabey T. B. Scott B. A. Lin S-H(J Koopman J. (2020). Not all fairness is created equal: A study of employee attributions of supervisor justice motives. Journal of Applied Psychology, 105(3), 274-293. 10.1037/apl0000440 31380668

[bibr59-15480518251352592] Matta F. K. Scott B. A. Colquitt J. A. Koopman J. Passantino L. G. (2017). Is consistently unfair better than sporadically fair? An investigation of justice variability and stress. Academy of Management Journal, 60(2), 743-770. 10.5465/amj.2014.0455

[bibr60-15480518251352592] Mayer R. C. Davis J. H. (1999). The effect of the performance appraisal system on trust for management: A field quasi-experiment. Journal of Applied Psychology, 84(1), 123-136. 10.1037/0021-9010.84.1.123

[bibr61-15480518251352592] Mayer R. C. Davis J. H. Schoorman F. D. (1995). An integrative model of organizational trust. Academy of Management Review, 20(3), 709-734. 10.2307/258792

[bibr62-15480518251352592] McAllister D. J. (1995). Affect-and cognition-based trust as foundations for interpersonal cooperation in organizations. Academy of Management Journal, 38(1), 24-59. 10.5465/256727

[bibr63-15480518251352592] Morgeson F. P. DeRue D. S. (2006). Event criticality, urgency, and duration: Understanding how events disrupt teams and influence team leader intervention. The Leadership Quarterly, 17(3), 271-287. 10.1016/j.leaqua.2006.02.006

[bibr64-15480518251352592] Morgeson F. P. Mitchell T. R. Liu D. (2015). Event system theory: An event-oriented approach to the organizational sciences. Academy of Management Review, 40(4), 515-537. https://doi.org/tps://doi.org/10.5465/amr.2012.0099

[bibr65-15480518251352592] MSN. (2021, December 14). MDHR sues McDonald’s franchisee for workplace sexual assault and harassment. https://www.msn.com/en-us/news/us/mdhr-sues-mcdonald-s-franchisee-for-workplace-sexual-assault-and-harassment/ar-AAROrro.

[bibr66-15480518251352592] Muthén L. K. Muthén B. O. (2018). Mplus user’s guide (8th Ed.). Muthén & Muthén.

[bibr67-15480518251352592] Organ D. W. (1988). Organizational citizenship behavior: The good soldier syndrome. Lexington Books.

[bibr68-15480518251352592] Pearce J. L. Gregersen H. B. (1991). Task interdependence and extrarole behavior: A test of the mediating effects of felt responsibility. Journal of Applied Psychology, 76(6), 838-844. 10.1037/0021-9010.76.6.838

[bibr69-15480518251352592] Petrin M. (2012). The curious case of directors’ and officers’ liability for supervision and management: Exploring the intersection of corporate and tort law. American University Law Review, 59(6), 1661-1711. 10.2139/SSRN.1407589

[bibr70-15480518251352592] Podsakoff P. M. MacKenzie S. B. Paine J. B. Bachrach D. G. (2000). Organizational citizenship behaviors: A critical review of the theoretical and empirical literature and suggestions for future research. Journal of Management, 26(3), 513-563. 10.1016/S0149-2063(00)00047-7

[bibr71-15480518251352592] Podsakoff P. M. MacKenzie S. B. Podsakoff N. P. (2012). Sources of method bias in social science research and recommendations on how to control it. Annual Review of Psychology, 63(1), 539-569. 10.1146/annurev-psych-120710-100452 21838546

[bibr72-15480518251352592] Podsakoff P. M. MacKenzie S. B. Podsakoff N. P. (2016). Recommendations for creating better concept definitions in the organizational, behavioral, and social sciences. Organizational Research Methods, 19(2), 159-203. 10.1177/1094428115624965

[bibr73-15480518251352592] Quade M. J. Bonner J. M. Greenbaum R. L. (2022). Management without morals: Construct development and initial testing of amoral management. Human Relations, 75(2), 273-303. 10.1177/0018726720972784

[bibr74-15480518251352592] Robinson S. L. (1996). Trust and breach of the psychological contract. Administrative Science Quarterly, 41(4), 574-599. 10.2307/2393868

[bibr75-15480518251352592] Rupp D. E. Cropanzano R. (2002). The mediating effects of social exchange relationships in predicting workplace outcomes from multifoci organizational justice. Organizational Behavior and Human Decision Processes, 89(1), 925-946. 10.1016/S0749-5978(02)00036-5

[bibr76-15480518251352592] Schoemann A. M. Boulton A. J. Short S. D. (2017). Determining power and sample size for simple and complex mediation models. Social Psychological and Personality Science, 8(4), 379-386. 10.1177/1948550617715068

[bibr77-15480518251352592] Shaw J. C. Wild E. Colquitt J. A. (2003). To justify or excuse?: A meta-analytic review of the effects of explanations. Journal of Applied Psychology, 88(3), 444-458. 10.1037/0021-9010.88.3.444 12814294

[bibr78-15480518251352592] Shoss M. K. Eisenberger R. Restubog S. L. D. Zagenczyk T. J. (2013). Blaming the organization for abusive supervision: The roles of perceived organizational support and supervisor's organizational embodiment. Journal of Applied Psychology, 98(1), 158-168. 10.1037/a0030687 23205496

[bibr79-15480518251352592] Skarlicki D. P. Barclay L. J. Pugh D. S. (2008). When explanations for layoffs are not enough: Employer's integrity as a moderator of the relationship between informational justice and retaliation. Journal of Occupational and Organizational Psychology, 81(1), 123-146. 10.1348/096317907X206848

[bibr80-15480518251352592] Skarlicki D. P. Kulik C. T. (2004). Third-party reactions to employee (mis) treatment: A justice perspective. Research in Organizational Behavior, 26, 183-229. 10.1016/S0191-3085(04)26005-1

[bibr81-15480518251352592] Spector P. E. (2021). Mastering the use of control variables: The hierarchical iterative control (HIC) approach. Journal of Business and Psychology, 36, 737-750. 10.1007/s10869-020-09709-0

[bibr82-15480518251352592] Stickley A. (2017). Illegality as a defence to negligence. Australian Civil Liability, 14(5), 73-75.

[bibr83-15480518251352592] Tepper B. J. Eisenbach R. J. Kirby S. L. Potter P. W. (1998). Test of a justice-based model of subordinates’ resistance to downward influence attempts. Group & Organization Management, 23(2), 144-160. 10.1177/1059601198232004

[bibr84-15480518251352592] Tsui A. S. (2021). Responsible research and responsible leadership studies. Academy of Management Discoveries, 7(2), 166-170. 10.5465/amd.2019.0244

[bibr85-15480518251352592] Van Dyne L. V. Ang S. Botero I. C. (2003). Conceptualizing employee silence and employee voice as multidimensional constructs. Journal of Management Studies, 40(6), 1359-1392. 10.1111/1467-6486.00384

[bibr86-15480518251352592] Watson D. Clark L. A. Tellegen A. (1988). Development and validation of brief measures of positive and negative affect: The PANAS scales. Journal of Personality and Social Psychology, 54(6), 1063-1070. 10.1037/0022-3514.54.6.1063 3397865

[bibr87-15480518251352592] Wright T. A. Bonett D. G. (2007). Job satisfaction and psychological well-being as nonadditive predictors of workplace turnover. Journal of Management, 33(2), 141-160. 10.1177/0149206306297582

[bibr88-15480518251352592] Wright T. A. Cropanzano R. (2000). Psychological well-being and job satisfaction as predictors of job performance. Journal of Occupational Health Psychology, 5(1), 84-94. 10.1037/1076-8998.5.1.84 10658888

[bibr89-15480518251352592] Yeung S. K. Yay T. Feldman G. (2022). Action and inaction in moral judgments and decisions: Meta-analysis of omission bias omission-commission asymmetries. Personality and Social Psychology Bulletin, 48(10), 1499-1515. 10.1177/01461672211042315 34496694

[bibr90-15480518251352592] Zhong R. Robinson S. L. (2021). What happens to bad actors in organizations? A review of actor-centric outcomes of negative behavior. Journal of Management, 47(6), 1430-1467. 10.1177/0149206320976808

